# Safety and efficacy of microbiota-directed adjunctive therapy in acute pancreatitis: a systematic review and meta-analysis of prebiotics, probiotics, and synbiotics

**DOI:** 10.3389/fmed.2026.1873327

**Published:** 2026-09-11

**Authors:** Ji Sun, Jun Zheng, Ye Shen

**Affiliations:** Department of Critical Care Medicine, Inner Mongolia People's Hospital, Affiliated to Baotou Medical College, Hohhot, China

**Keywords:** acute pancreatitis, efficacy, hospital stay, meta-analysis, microbiota, mortality, prebiotics, probiotics

## Abstract

**Background:**

Prebiotics, probiotics, and synbiotics have been proposed as adjunctive treatments for acute pancreatitis (AP), but their clinical efficacy and safety remain uncertain. We conducted a systematic review and meta-analysis to evaluate their effects on recovery-related, efficacy, and safety-critical outcomes in AP.

**Methods:**

PubMed, Web of Science, Scopus, Embase, and the Cochrane Library were searched through July 2026, supplemented by Google Scholar and reference-list screening. Comparative interventional and observational studies evaluating prebiotics, probiotics, or synbiotics versus placebo, standard care, or nutritional controls were eligible. Risk of bias was assessed using Cochrane RoB 2 for randomized studies and the Newcastle–Ottawa Scale for non-randomized studies. Random-effects meta-analyses were performed using mean differences (MDs) or risk ratios (RRs) with 95% confidence intervals (CIs). Sensitivity and subgroup analyses were conducted to explore study quality and clinical heterogeneity.

**Results:**

30 studies were included in the systematic review and 25 in the meta-analysis. Microbiota-directed interventions were associated with a shorter hospital stay in the full-analysis set (MD − 4.68 days, 95% CI − 6.22 to −3.14), although heterogeneity was substantial (I^2^ = 98.74%); the association persisted in the sensitivity analysis (MD − 3.90 days, 95% CI − 5.93 to −1.87). Mortality was lower in the full analysis (RR 0.67, 95% CI 0.45–0.98), but this finding was not robust and lost statistical significance in the sensitivity analysis. Total infection (RR 0.50, 95% CI 0.36–0.69) and infectious morbidity (RR 0.58, 95% CI 0.42–0.80) were reduced, whereas infected pancreatic necrosis, organ failure/multiple organ failure, need for operation, and systemic inflammatory response syndrome were not significantly improved overall. Adverse-event reporting was inconsistent, and a large multicenter trial provided an important safety signal for live multispecies probiotics in predicted severe AP.

**Conclusion:**

Microbiota-directed interventions may be associated with shorter hospital stay and fewer infection-related complications, but substantial heterogeneity, intervention diversity, and incomplete safety reporting limit clinical interpretation. Evidence for mortality or other severe outcomes is not robust. Current evidence does not support routine use of these interventions in AP, particularly live probiotic preparations in severe or critically ill patients. Future adequately powered, severity-stratified trials using standardized formulations and prospective safety monitoring are needed.

**Systematic review registration:**

Registration number: CRD420261387279.

## Introduction

Acute pancreatitis (AP) is an inflammatory disorder of the pancreas with a clinical spectrum ranging from mild, self-limiting disease to severe acute pancreatitis (SAP), which is associated with pancreatic necrosis, systemic inflammatory response syndrome (SIRS), organ failure, sepsis, prolonged hospitalization, and increased mortality (1, 2).

In severe disease, local pancreatic injury is amplified by systemic inflammation, impaired pancreatic microcirculation, and intestinal barrier dysfunction. Disruption of the gut barrier may facilitate bacterial and endotoxin translocation into the circulation and necrotic pancreatic tissue, thereby increasing the risk of infected necrosis and multiorgan complications (3–5).

Prebiotics, probiotics, and synbiotics have therefore been proposed as adjunctive strategies to modulate intestinal microbiota, support mucosal barrier integrity, and reduce microbial translocation. Probiotics are live microorganisms intended to confer host benefit, prebiotics are substrates that promote beneficial bacteria, and synbiotics combine both approaches to potentially enhance restoration of intestinal microbial balance (6).

However, the clinical value and safety of microbiota-directed therapy in AP remain uncertain. Individual studies and prior reviews have reported heterogeneous findings, with possible reductions in recovery-related outcomes such as hospital stay but inconsistent effects on mortality, organ failure, infectious complications, and other clinically critical endpoints (2, 3, 5, 6). These uncertainties, together with concerns about probiotic use in severely ill patients, support the need for an updated synthesis of available evidence.

Several clinical studies have investigated the potential benefits of microbiota-modulating therapies in patients with acute pancreatitis. Early clinical trials demonstrated that probiotic supplementation could improve intestinal barrier function and reduce inflammatory markers in patients with severe acute pancreatitis. For example, the use of gentamicin combined with probiotics was shown to significantly reduce levels of inflammatory markers such as procalcitonin, interleukin-6, and C-reactive protein, suggesting improved infection control and intestinal barrier protection (7). Similarly, supplementation with Bifidobacterium in combination with early enteral nutrition has been associated with improved nutritional status, enhanced immune function, and reduced hospital stay in patients with severe acute pancreatitis (8).

More recent studies have explored combined therapeutic strategies involving probiotics and pharmacological or nutritional interventions. For instance, probiotics administered alongside ulinastatin and somatostatin were found to significantly reduce inflammatory factors and improve clinical outcomes compared with standard therapy alone (1). Likewise, early micro-ecological enteral nutrition has been shown to decrease bacterial translocation, restore intestinal immune function, and reduce complications in patients with severe acute pancreatitis (9).

Emerging evidence has also highlighted the potential benefits of novel combination therapies. A recent randomized clinical study demonstrated that the combination of probiotics with herbal therapy significantly improved inflammatory markers, intestinal barrier function, and clinical recovery in patients with severe acute pancreatitis, suggesting a synergistic therapeutic effect (10). These findings indicate that microbiota-based therapies may play an important role in improving outcomes in acute pancreatitis.

Despite these promising results, the clinical effectiveness and safety of pre-, pro-, and synbiotics in acute pancreatitis remain controversial. Some studies have reported beneficial effects on infection rates, immune function, and hospitalization duration, whereas others have demonstrated no significant improvement in major clinical outcomes such as mortality or organ failure. For example, a retrospective analysis of probiotic prophylaxis in patients with predicted severe acute pancreatitis found no significant positive or negative impact on infectious complications or mortality, highlighting the uncertainty surrounding probiotic therapy in this patient population (11).

Previous meta-analyses have evaluated microbiota-based interventions in acute pancreatitis, but many focused primarily on probiotics or broad pooled effects and provided limited stratification by disease severity, intervention timing, formulation, strain or substrate composition, and safety profile. The present review adds to the existing literature by updating the evidence base; evaluating prebiotic, probiotic, and synbiotic interventions separately and collectively; exploring clinically relevant sources of heterogeneity; and systematically summarizing safety-critical outcomes and study-reported adverse events.

Given the increasing number of clinical trials evaluating microbiota-based therapies and the persistent uncertainty regarding their safety and efficacy, an updated synthesis of available evidence is warranted. In particular, concerns regarding potential adverse outcomes associated with probiotic therapy underscore the need for careful evaluation of both therapeutic benefits and safety profiles in patients with acute pancreatitis.

Therefore, the present systematic review and meta-analysis aim to comprehensively evaluate the safety and efficacy of pre-, pro-, and synbiotics in patients with acute pancreatitis by synthesizing evidence from randomized controlled trials and clinical studies.

## Methods

In this systematic review, we followed the Preferred Reporting Items for Systematic Reviews and Meta-Analyses (PRISMA) guidelines (12). We also registered this review in PROSPERO (registration number: CRD420261387279). The review timeline started in February 2026. The final analysis was consistent with the registered protocol regarding PICOS, eligibility criteria, and the pre-specified primary and secondary outcomes.

### Literature search and study selection

A comprehensive literature search was conducted up to July 2026 across the following databases: PubMed, Web of Science, Scopus, Embase, and Cochrane. We applied no language restrictions to our initial search. This search was further supplemented by a manual search in Google Scholar and reference lists of included studies and previously published review articles to identify potentially missed studies. Detailed keywords and search strategies for each database are available in Supplementary Tables 1, 2.

**Figure 1:**
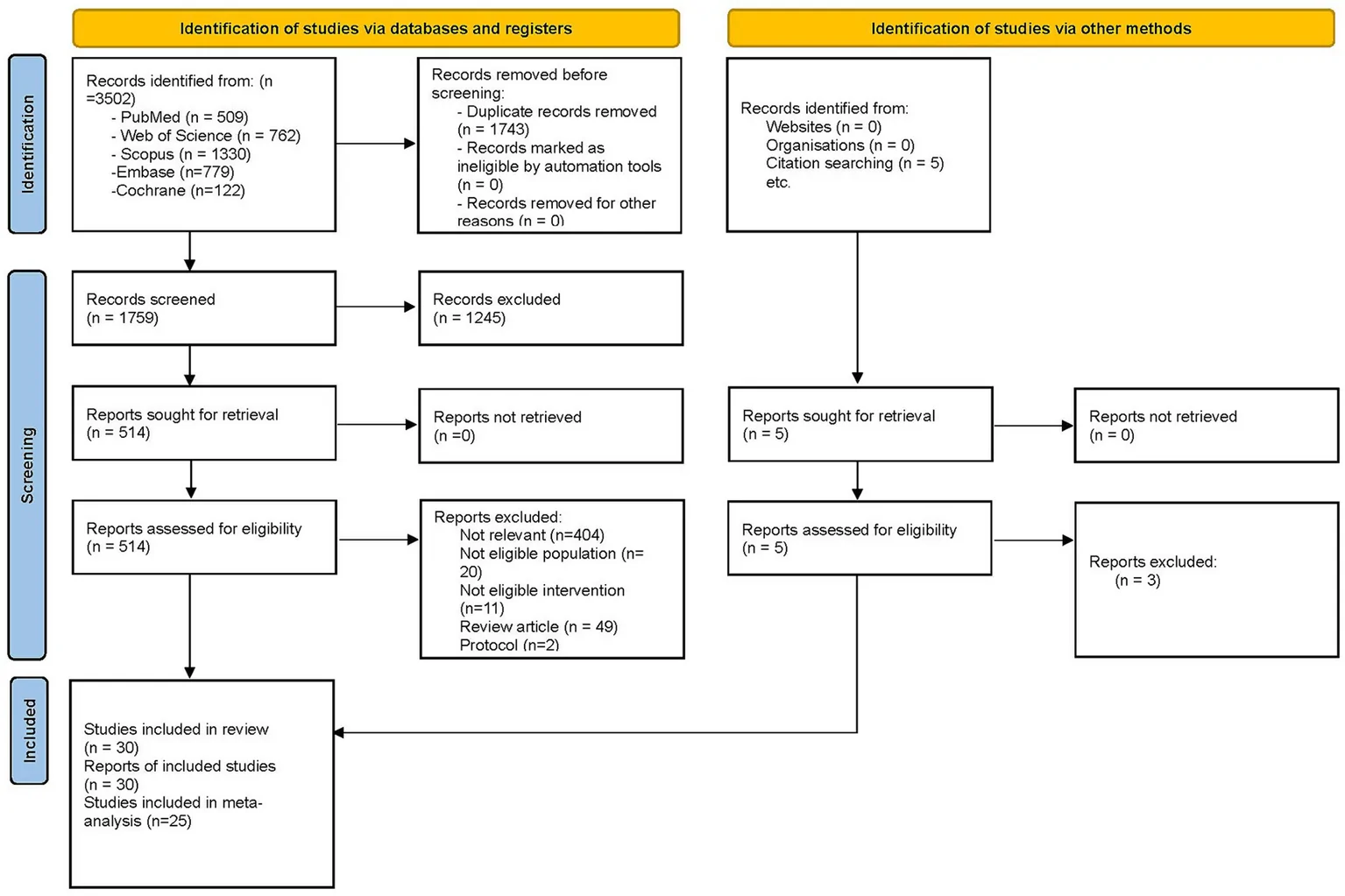
PRISMA flow diagram indicating identification, and screening process and final included studies.

After removing duplicates, to reduce the possibility of excluding potentially pertinent research, two independent reviewers (JZ and YS) screened the titles and abstracts of the identified records. Full-text articles were then assessed for inclusion, with any disagreements resolved through discussion or by consulting a third reviewer (JS). Case reports, qualitative studies, and reviews were all excluded. The PRISMA flowchart outlining the selection process is presented in Figure 1.

### Inclusion criteria

The studies included in this systematic review met the following criteria: (1) observational or interventional study designs including cohort studies, and clinical trials, (2) published in peer-reviewed journals, (3) involving patients with acute pancreatitis, with a focus on prebiotics, probiotics or synbiotics as an intervention versus placebo or standard treatment, and (4) reporting data on the efficacy or safety of interventions. Table 1 shows a dedicated PICOS framework summarizing the eligibility criteria, including study population, eligible microbiota-directed interventions, comparator categories, primary and secondary outcomes, safety outcomes, and eligible study designs.

**Table 1:** PICOS framework of the systematic review and meta-analysis.

PICOS domain	Definition in this review	Operational details
Population	Patients diagnosed with acute pancreatitis	Adult populations with acute pancreatitis were eligible, regardless of etiology. Studies including mild, moderately severe, severe, or predicted severe acute pancreatitis were included. Disease severity was extracted and categorized as mild/mild-to-moderate, severe/predicted severe, mixed, or unclear when not sufficiently reported.
Intervention	Microbiota-directed adjunctive interventions	Eligible interventions included prebiotics, probiotics, and synbiotics used as adjunctive therapy in acute pancreatitis. Intervention details were extracted when available, including intervention class, specific probiotic strain or genus, prebiotic substrate, synbiotic formulation, single- versus multi-strain status, dose, formulation, route of administration, timing of initiation, duration of treatment, and whether the intervention was administered with enteral nutrition or another nutritional protocol.
Comparator	Placebo, standard care, or nutritional control	Eligible comparators included placebo, standard acute pancreatitis care, standard enteral nutrition, parenteral nutrition, or other non-microbiota-directed nutritional/supportive regimens. Comparator category and background nutritional therapy were extracted because these factors may modify clinical outcomes, particularly hospital stay and infection-related outcomes.
Outcomes	Recovery-related, efficacy-related, and safety-critical outcomes	The primary outcomes were length of hospital stay and mortality. Secondary outcomes included total infection or infectious morbidity, infected pancreatic necrosis, organ failure or multiple organ failure, systemic inflammatory response syndrome, need for operation or invasive intervention, and other reported clinical complications. Safety outcomes included mortality, infection-related complications, bowel or intestinal ischemia, bacteremia, fungemia or probiotic-related infection, gastrointestinal intolerance, diarrhea, vomiting, lactic acidosis, sepsis, and other adverse events reported by individual studies.
Study design	Comparative clinical studies	Randomized controlled trials and non-randomized comparative clinical studies were eligible. Single-arm studies, case reports, reviews, editorials, animal studies, and *in vitro* studies were excluded from quantitative synthesis. Study design was extracted and used in subgroup or sensitivity analyses where applicable.

### Data extraction

Initially, relevant data were extracted by two authors (JZ and YS) and subsequently verified by a third author (JS). This data collection followed a prepared checklist that included individual patient details, such as first author, publication year, country, type of study, type of intervention, intervention and control regimen, sample size, age and gender in each group, baseline characteristics of patients (e.g., APACHE score, etc.), and baseline CRP level. We also extracted data on hospital stay and mortality as primary outcomes and infection of necrotic pancreas tissue, total infection, organ failure/MOF, required operation, infectious morbidity, and SIRS as secondary outcomes, where available. It should be noted that not all studies reported data on all above outcomes, but we extracted as much data as provided by each single individual study to perform a comprehensive meta-analysis. To better address clinical heterogeneity, we additionally extracted study-level characteristics relevant to subgroup analysis, including acute pancreatitis severity, specific probiotic strain, single- versus multi-strain formulation, route and pharmaceutical/nutritional formulation, timing of initiation, treatment duration, and comparator category. When specific strains were not reported in sufficient detail, these variables were coded as unclear.

### Quality assessment

Risk of bias was assessed at the study level using design-specific instruments. Randomized studies were evaluated with the Cochrane Risk of Bias 2 (RoB 2) tool, and non-randomized comparative studies were assessed with the Newcastle–Ottawa Scale (NOS). Judgments were made conservatively when methodological reporting was incomplete.

For randomized studies, RoB 2 assessments covered the domains of bias arising from the randomization process, deviations from intended interventions, missing outcome data, measurement of the outcome, and selection of the reported result. Each domain was judged as low risk, some concerns, or high risk, and an overall study-level judgment was then assigned according to standard RoB 2 principles (13).

For non-randomized studies, NOS assessments addressed representativeness of the exposed cohort, source of the comparison cohort, ascertainment of exposure, confirmation that outcomes were not present at baseline, comparability of groups, outcome assessment, adequacy of follow-up duration, and completeness of follow-up. Star-based NOS judgments were translated into overall low-, moderate-, or high-risk categories, with particular emphasis placed on control of confounding. Separate traffic-light summaries were prepared for randomized and non-randomized studies (14).

### Statistical analysis

Meta-analysis was performed in Stata using a random-effects framework because substantial clinical and methodological heterogeneity was anticipated across study designs, intervention formulations, comparators, severity definitions, and outcome ascertainment. Dichotomous outcomes were synthesized as risk ratios (RRs) with 95% confidence intervals, and continuous outcomes were synthesized as mean differences (MDs) with 95% confidence intervals. Hospital stay was treated as the principal continuous outcome. Where necessary, nonparametric summaries that had been transformed to approximate means and standard deviations for pooling were retained in the final quantitative dataset and explicitly identified in the descriptive table. When studies reported continuous data as medians with interquartile ranges, means and standard deviations were estimated using the method described by Wan et al., based on the reported median, first and third quartiles, and sample size (15).

For continuous outcomes, random-effects models were estimated using restricted maximum likelihood (REML). For dichotomous outcomes, random-effects models were also used; depending on the specific Stata command and final exported model, between-study variance was estimated using REML or the DerSimonian–Laird method, as indicated on the corresponding forest plots. Statistical heterogeneity was assessed using Cochran’s Q test, the I^2^ statistic, and τ^2^. Forest plots were generated for all pooled outcomes.

Subgroup analyses were conducted for all outcomes according to intervention category (prebiotic, probiotic, or synbiotic). For the two primary outcomes, additional subgroup analyses were performed according to study design (RCT vs. non-RCT). In response to the substantial heterogeneity observed for hospital stay and to improve clinical interpretability, we also performed additional exploratory subgroup analyses according to disease severity, timing of intervention initiation, intervention duration, comparator category, formulation category, single- versus multi-strain formulation, and the presence or absence of major administered genera where extractable from the full texts. These genera included Lactobacillus, Bifidobacterium, Bacillus, Enterococcus, Lactococcus, and Streptococcus. These analyses were considered exploratory because several subgroups included only a small number of studies and because reporting of strain composition, dose, timing, and co-interventions was inconsistent across trials.

We considered network meta-analysis but did not perform it because the available evidence did not form a sufficiently connected or clinically transitive network. Specific strains, and commercial formulations were often represented by single studies; comparators varied substantially across placebo, standard enteral nutrition, parenteral nutrition, and standard care; and disease severity and timing of intervention were inconsistently reported. Under these conditions, indirect comparisons would likely be unstable and potentially misleading.

A secondary sensitivity dataset was analyzed separately after excluding studies not retained in the final sensitivity model, and the same meta-analytic framework was then repeated for all available outcomes. Leave-one-out influence analyses were performed for the primary outcomes to evaluate the influence of each individual study on the pooled estimate.

Potential small-study effects/publication bias were explored only for the primary outcomes. Funnel plots were examined visually, and Egger’s regression test and Begg’s rank-correlation test were performed when data were sufficient. Nonparametric trim-and-fill analyses were additionally undertaken for hospital stay and mortality; these results were interpreted cautiously when convergence warnings were generated.

## Results

A total of 3,502 records were identified through database searching, including 509 from PubMed, 762 from Web of Science, 1,330 from Scopus, 779 from Embase, and 122 from the Cochrane Library. An additional 5 records were identified through citation searching. After removal of 1,743 duplicate records, 1,759 records underwent title and abstract screening, of which 1,245 were excluded. A total of 514 reports identified through database searching were sought for retrieval, and all were successfully retrieved and assessed for eligibility. Of these, 486 reports were excluded. In addition, all 5 reports identified through citation searching were retrieved and assessed for eligibility, of which 3 were excluded. Ultimately, 30 studies were included in the systematic review, and 25 provided sufficient data for inclusion in the meta-analysis. Ultimately, 30 studies (1, 7, 8, 10, 16–41) were included in the systematic review, of which 25 (8, 16–20, 22–33, 35–41) provided sufficient data for inclusion in the meta-analysis (Figure 1).

### Study characteristics

Table 2 summarizes 31 comparison-level entries derived from 30 studies, because the Plaudis (40) study contributed separate prebiotic and synbiotic comparisons. Most included studies were randomized controlled trials (*n* = 25), while a smaller number were prospective feasibility studies, retrospective cohorts, retrospective comparative analyses, or other comparative clinical studies. The evidence base was dominated by probiotic interventions, with only a few prebiotic and synbiotic comparisons. Geographically, the literature was heavily concentrated in China (1, 7–10, 18–30, 33–35, 37, 39), with additional studies from Turkey (41), Latvia (40), Hungary (17, 38), Netherlands (36), the Czech Republic (32), and India (16, 31).

**Table 2:** Characteristics of included studies.

No.	First author (Year) [Country]	Study type	Intervention type	Intervention regimen	Control regimen	Sample size, age, gender (I/C)	Baseline characteristics CRP (I/C)
1	Karakan (2007) (41) [Turkey]	RCT	Prebiotic	Prebiotic fiber-supplemented enteral nutrition	Standard enteral solution	*n*: 15/15; age: 47.3 ± 16.8/44.9 ± 11.2; M/F: I:6/9; C:8/7	APACHE II: 9.4 ± 3.7/9.6 ± 3.8; Ranson: NR; CRP: 232 ± 97/244 ± 104
2	Plaudis (2012) (40) [Latvia]	Prospective feasibility study	Prebiotic	Prebiotic-supplemented low-volume enteral stimulation (prebiotic arm)	Low-volume enteral stimulation control	*n*: 28/32; age: NR; M/F: NR	APACHE II: 8.8/8.6; Ranson: NR; CRP: NR
3	Liu (2025) (39) [China]	Retrospective cohort	Prebiotic	Early enteral nutrition + fructooligosaccharides (FOS)	Standard early enteral nutrition	*n*: 37/73; age: 48.19 ± 14.65/47.23 ± 14.73; M/F: I:24/13; C:51/22	APACHE II: 10/12; NRS-2002 score: 4/4; SOFA score: 5/6; CRP: 192/254
4	Oláh (2002) (38) [Hungary]	Clinical trial	Probiotic	Live *Lactobacillus plantarum* 299 + oat fiber	Heat-killed *Lactobacillus plantarum* 299 + oat fiber	*n*: 22/23; age: 44.1 ± 11.1/46.5 ± 13.6; M/F: I:16/6; C:17/6	Glasgow score: 2.5/2.8; CRP: 206/188
5	Li (2007) (37) [China]	Non-RCT	Probiotic	Traditional treatment + probiotics (Jinshuangqi)	Traditional treatment only	*n*: 14/11; age: 45 ± 13/NR; M/F: NR	NR
6	Besselink (2008) (36) [Netherlands]	RCT	Probiotic	Ecologic 641 multispecies probiotic	Placebo	*n*: 152/144; age: 60.4 ± 16.5/59 ± 15.5; M/F: I:91/61; C:83/61	APACHE II: 8.6/8.4; Imrie score: 3.3/3.4; SOFA score: 2.1/1.9; CRP: 268/270
7	Qin (2008) (35) [China]	RCT	Probiotic	*Lactobacillus plantarum* enteral feeding + parenteral nutrition	Parenteral nutrition	*n*: 36/38; age: 54.3 ± 13.1/58.4 ± 19.1; M/F: I:11/25; C:12/26	APACHE II: 8.8/8.9; Balthazar CT score: 4.7/4.5; CRP: 125/136
8	Cui (2009) (34) [China]	RCT	Probiotic	Early enteral probiotics (EEP)	Early enteral nutrition alone (EEN)	*n*: 20/25; age: 45.3/NR; M/F: NR	NR
9	Wu (2009) (33) [China]	Non-RCT	Probiotic	Probiotic adjuvant therapy	Control	*n*: 14/13; age: NR; M/F: NR	NR
10	Lata (2010) (32) [Czech Republic]	RCT	Probiotic	Six-strain probiotic preparation	Placebo	*n*: 7/15; age: 52 ± 12/55 ± 13; M/F: I:3/4; C:10/5	NR
11	Sharma (2011) (31) [India]	RCT	Probiotic	Probiotics (4 sachets/day for 7 days)	Placebo	*n*: 24/26; age: 41 ± 20.72/40.19 ± 17.43; M/F: I:12/12; C:11/15	APACHE II: 5.45 ± 5.5/4.84 ± 4.23; CRP: 275/228
12	Cui (2013) (30) [China]	RCT	Probiotic	Early enteral nutrition + probiotics (P + EN)	Enteral nutrition alone (EN)	*n*: 23/25; age: NR; M/F: NR	NR
13	Wang (2013) (29) [China]	RCT	Probiotic	Enteral nutrition + ecoimmunonutrition (EN + EIN)	Enteral nutrition alone (EN)	*n*: 62/61; age: 42.6 ± 13.8/41.7 ± 11.4; M/F: I:32/30; C:34/27	APACHE II: 12.88 ± 3.19/14.63 ± 3.67/13.27 ± 2.86
14	Li (2014) (28) [China]	RCT	Probiotic	Enteral nutrition + Bifico (P + EN)	Enteral nutrition alone (EN)	*n*: 27/28; age: 49.3 ± 11.5/47.5 ± 9.6; M/F: I:17/10; C:17/11	Ranson: 5.1 ± 1.7/4.9 ± 2.0; APACHE II: 11.0 ± 2.0/11.1 ± 2.7; CT grade D/E 14/13 vs. 13/15
15	Zhu (2014) (27) [China]	RCT	Probiotic	*Clostridium butyricum* probiotic	Placebo (starch)	*n*: 20/19; age: 43.5 ± 17.5/42 ± 16.5; M/F: I:11/9; C:10/9	NR
16	Liu (2015) (26) [China]	Retrospective analysis	Probiotic	Enteral nutrition + probiotics	Enteral nutrition alone	*n*: 47/32; age: 47.3 ± 10.8/48.5 ± 13.7; M/F: I:32/15; C:21/11	APACHE II: 11.4 ± 2.9/10.8 ± 3.1; CRP: 349/364
17	Wang (2017) (7) [China]	RCT	Probiotic	Gentamicin combined with probiotics	Routine/basic treatment	*n*: 35/36; age: NR; M/F: I:22/NR; C:20/NR	CRP: 192.34 ± 5.25/189.72 ± 8.14
18	Wu (2017) (25) [China]	RCT	Probiotic	Probiotics + early enteral nutrition + routine treatment	Routine treatment	*n*: 60/60; age: 42.7 ± 11.5/42.6 ± 13.6; M/F: I:34/26; C:32/28	APACHE II: 10.8 ± 2.9/11.3 ± 3.1; Ranson: 5.1/4.9; CRP: NR
19	Fang (2018) (24) [China]	RCT	Probiotic	Antibiotics + probiotics	Antibiotics alone	*n*: 34/34; age: 46.37 ± 10.82/35.91 ± 11.04; M/F: I:22/12; C:24/10	APACHE II: 10.79 ± 3.02/11.04 ± 2.89; CRP: 191/189
20	Jin (2018) (8) [China]	Retrospective pilot study	Probiotic	Bifidobacterium + early enteral nutrition	Early enteral nutrition alone	*n*: 30/30; age: 46.31 ± 11.23/45.38 ± 12.23; M/F: I:19/11; C:17/13	NR
21	Liao (2020) (23) [China]	RCT	Probiotic	Probiotics + enteral nutrition	Enteral nutrition alone	*n*: 50/50; age: 48.7 ± 10.5/49.1 ± 10.3; M/F: I:29/21; C:31/19	APACHE II: 11.2 ± 3.1/11.5 ± 3; CRP: 191/190
22	Wan (2021) (22) [China]	RCT	Probiotic	Probiotic capsules (*Bacillus subtilis* + *Enterococcus faecium*)	Placebo capsules	*n*: 64/64; age: 50.25 ± 16.79/54.72 ± 14.86; M/F: I:40/24; C:42/22	Charlson score: 3.02 ± 1.12/2.96 ± 0.29; CRP: 57/52
23	Xiao (2022) (21) [China]	RCT	Probiotic	Early enteral nutrition + microecological preparation	Early enteral nutrition alone	*n*: 40/40; age: 49 ± 10.93/44.19 ± 11.27; M/F: I:26/14; C:27/13	CRP: 320/321
24	Dou (2024) (1) [China]	Retrospective study	Probiotic	Probiotics + ulinastatin + somatostatin	Ulinastatin + somatostatin	*n*: 82/78; age: 42.89 ± 11.36/44.31 ± 10.57; M/F: I:51/31; C:43/35	CRP: 24/24
25	Zhao (2024) (20) [China]	RCT	Probiotic	Early feeding + Bifidobacterium quadruplex live bacterial tablets	Standard enteral nutrition / control	*n*: 34/32; age: 45.4 ± 2.28/44.65 ± 2.51; M/F: I:20/14; C:21/11	APACHE II: NR; BISAP score: 3.34 ± 0.19/2.96 ± 0.20; CRP: 151/156
26	Ma (2025) (10) [China]	RCT	Probiotic	Probiotics alone (Chang Le Kang)	Basic treatment only	*n*: 30/30; age: 49.63 ± 15.83/50.7 ± 15.76; M/F: I:22/8; C:17/13	APACHE II: 18.100 ± 4.715/19.470 ± 4.392; probiotic arm vs. control; CRP: 152.807/152.643
27	Cheng (2026) (19) [China]	RCT	Probiotic	Probiotics + enteral nutrition	Placebo + enteral nutrition	*n*: 65/65; age: 48.31 ± 5.43/47.98 ± 5.36; M/F: I:33/32; C:35/30	APACHE II: 15.31 ± 1.52/15.42 ± 1.58; CRP: 160/160
28	He (2026) (18) [China]	RCT	Probiotic	Saccharomyces boulardii + enteral nutrition	Enteral nutrition alone	*n*: 27/23; age: 47.33 ± 12.04/52.04 ± 13.36; M/F: I:19/8; C:15/8	APACHE II: 9/10; Marshall score: 2/4
29	Oláh (2007) (17) [Hungary]	RCT	Synbiotic	Synbiotic 2000 + prebiotic fibers	Prebiotic fibers only	*n*: 22/23; age: 53 ± 14/54 ± 13; M/F: I:16/6; C:17/6	APACHE II: 11.2 ± 3.1/11.4 ± 3.3; CRP: 230/240
30	Plaudis (2012) (40) [Latvia]	Prospective feasibility study	Synbiotic	Synbiotic/prebiotic-supplemented low-volume enteral stimulation (synbiotic arm)	Low-volume enteral stimulation control	*n*: 30/32; age: NR; M/F: NR	APACHE II: 8.8/8.6; Ranson: NR; CRP: NR
31	Rohith (2022) (16) [India]	RCT	Synbiotic	Synbiotic 1 g twice daily for 14 days	Placebo	*n*: 39/37; age: NR; M/F: I:31/8; C:31/6	APACHE II: 8.8/9; SOFA score: 3.1/3.9; Modified Marshall score: 1.8/2.4

Values are presented as intervention/control unless otherwise stated; Plaudis (40) is listed twice because synbiotic and prebiotic arms were extracted separately. APACHE II, Acute Physiology and Chronic Health Evaluation II; BISAP, Bedside Index for Severity in Acute Pancreatitis; C, control; CRP, C-reactive protein; d, days; EIN, ecoimmunonutrition; EN, enteral nutrition; F, female; FOS, fructooligosaccharides; HS, hospital stay; I, intervention; I/C, intervention/control; IM, infectious morbidity; IPN, infection of pancreatic necrosis; IQR, interquartile range; M, male; MOF, multiple organ failure; N/A, not applicable; NR, not reported; OF, organ failure; RCT, randomized controlled trial; RO, required operation; SD, standard deviation; SIRS, systemic inflammatory response syndrome; TI, total infection.

The interventions were clinically heterogeneous. Prebiotic strategies included prebiotic fiber-supplemented enteral formulas and fructooligosaccharide-supplemented enteral nutrition. Probiotic regimens ranged from single-strain preparations such as *Lactobacillus plantarum*, *Clostridium butyricum*, or Saccharomyces boulardii to multispecies or commercially available combinations such as Bifico, Jinshuangqi, *Bacillus subtilis* plus *Enterococcus faecium* capsules, and other microecological formulations. Synbiotic studies combined probiotic organisms with prebiotic fiber components. Comparators also varied substantially and included placebo, standard enteral nutrition, parenteral nutrition, routine/basic treatment, antibiotic-only regimens, and prebiotic-only control regimens. Treatment duration, when reported, generally ranged from 7 to 15 days (1, 8, 10, 16–19, 21, 26, 30, 31, 33, 35, 37, 38, 41), although some studies continued treatment until discharge (22) and one large multicenter trial administered treatment for 28 days (36).

Sample sizes also varied markedly, from very small single-center studies such as Lata (32) (7 vs. 15 participants) and Li (37) (14 vs. 11 participants) to the large multicenter Besselink (36) trial (152 vs. 144 participants). Most studies enrolled middle-aged adults, and the sex distribution was often reasonably balanced, although reporting was incomplete in several older or smaller studies. Baseline severity was assessed using a range of instruments, most commonly APACHE II (10, 16–20, 23–26, 29, 31, 35, 36, 39–41), with some studies also reporting Ranson score (25, 28, 40, 41), SOFA score (36, 39), BISAP score (20), Glasgow score (38), Marshall score (16, 18), Balthazar CT score (35), or modified CT severity indices. Baseline inflammatory burden was frequently represented by CRP (1, 7, 10, 17, 19–24, 26, 31, 35, 36, 38, 39, 41), but CRP was not uniformly reported across all studies (8, 16, 18, 25, 27–30, 32–34, 37, 40).

Outcome reporting was inconsistent across studies. Hospital stay was among the most frequently reported outcomes, and mortality was reported in many trials, whereas total infection, infected pancreatic necrosis, infectious morbidity, organ failure or multiple organ failure, SIRS, and need for operation were reported more selectively. Some studies reported outcomes as exact event counts suitable for meta-analysis, whereas others provided only partial or non-standardized reporting, including medians without standard deviations or incompletely defined infection outcomes. This variability in design, intervention content, comparator choice, and outcome definition supported the use of sub grouped and sensitivity-based quantitative syntheses.

### Quality assessment

**Figure 2:**
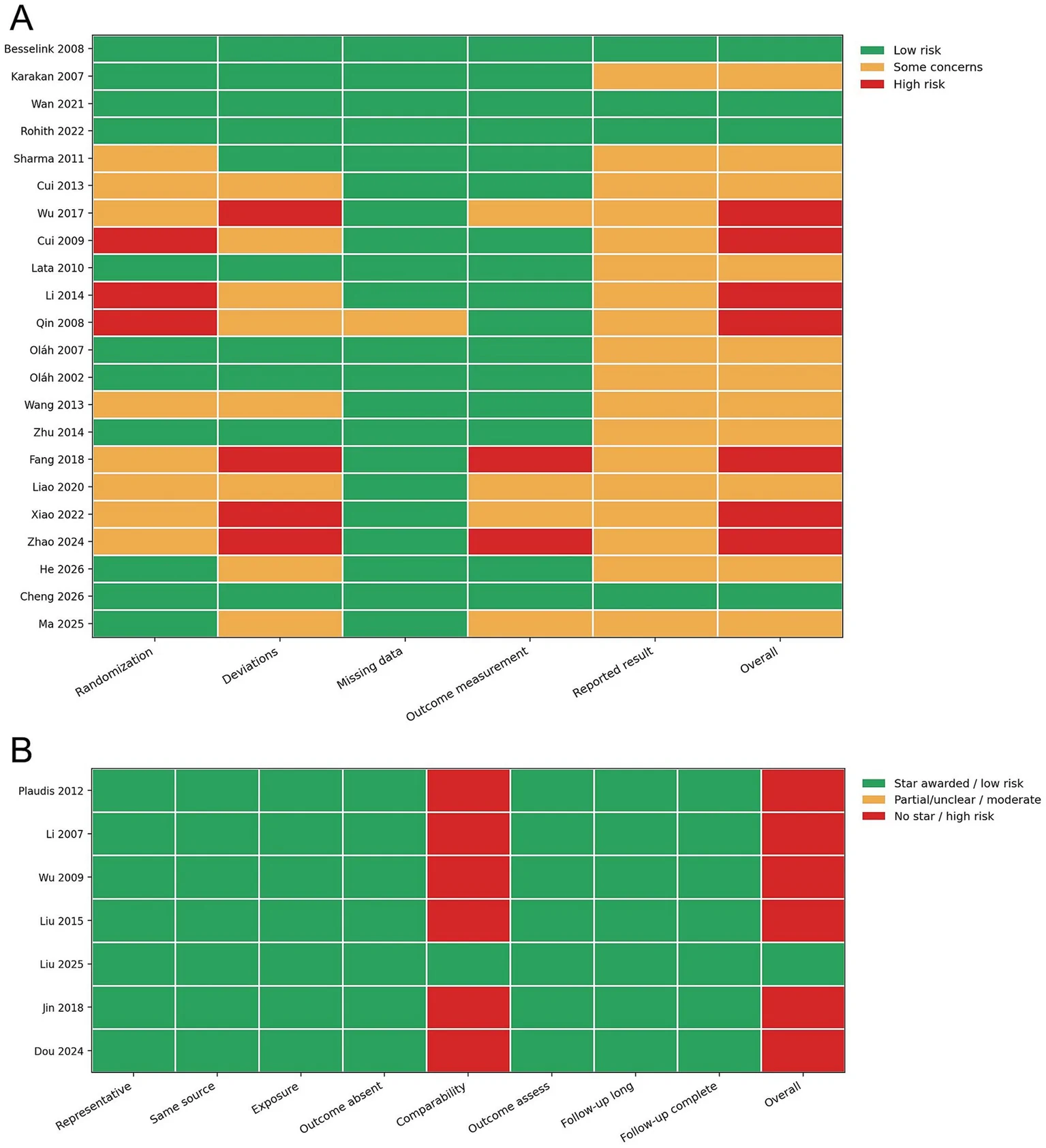
Quality assessment of included studies. **(A)** Traffic-light plot for randomized controlled trials assessed using the Cochrane Risk of Bias 2 tool. **(B)** Traffic-light plot for non-randomized studies assessed using the Newcastle–Ottawa Scale–based framework.

Risk of bias was assessed separately according to study design. Among randomized studies, several trials were judged as low risk of bias, whereas others raised some concerns or were rated high risk because of limited reporting of allocation procedures, absence of blinding, open-label designs, or outcome subjectivity. Non-randomized studies were generally at higher risk of bias, mainly because of non-random allocation and limited control for confounding. The detailed assessments are shown in Figure 2 and Supplementary Tables 3, 4.

### Primary outcomes

#### Length of hospital stay

**Figure 3:**
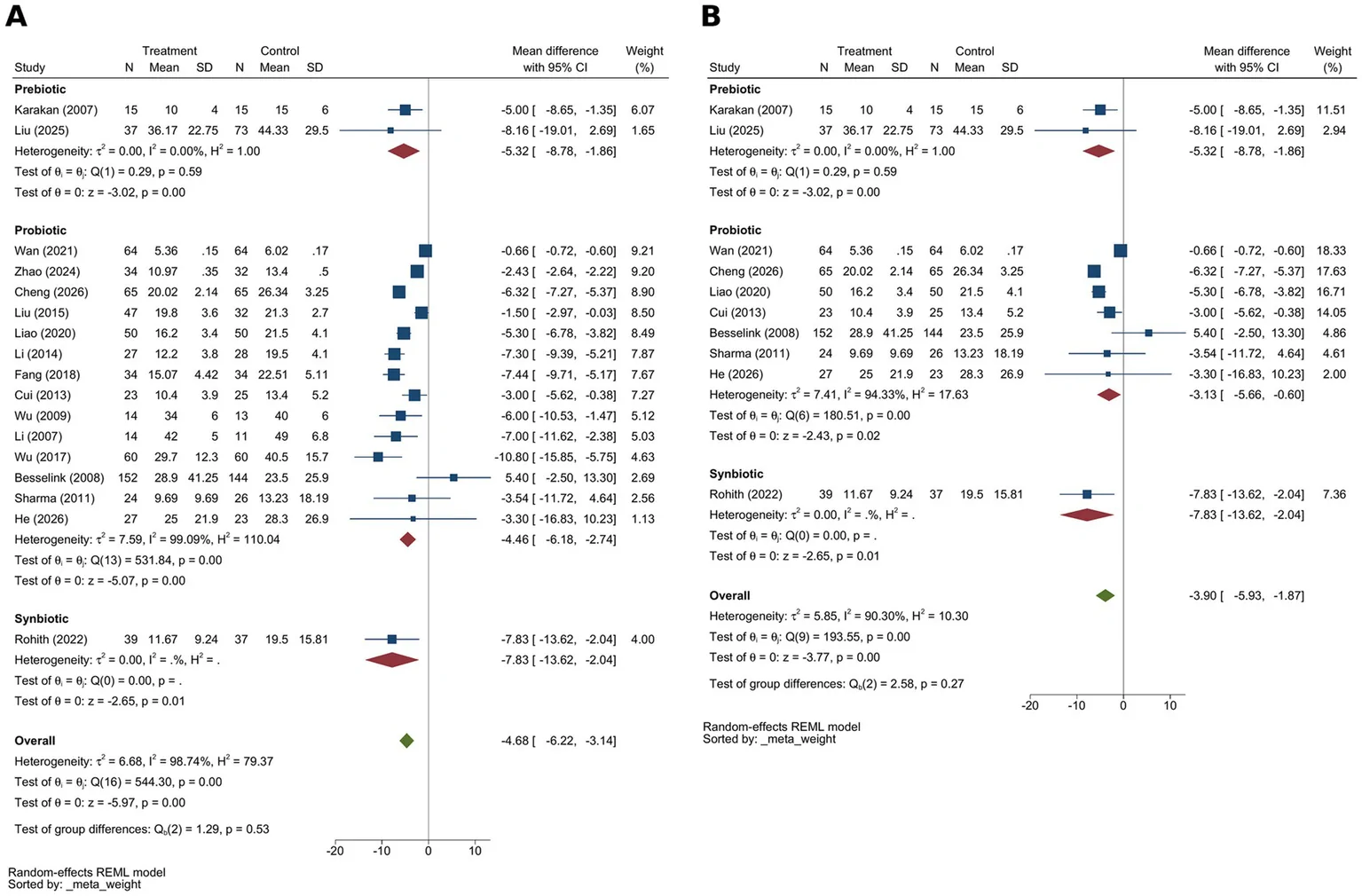
Effect of pre-, pro-, and synbiotics on hospital stay in acute pancreatitis. **(A)** Forest plot of the full-analysis set, subgrouped by intervention type (prebiotic, probiotic, and synbiotic). **(B)** Forest plot of the sensitivity-analysis set, subgrouped by intervention type. Negative mean differences favor microbiota-directed interventions.

In the full-analysis set, microbiota-directed interventions were associated with a significant reduction in hospital stay compared with control treatment (MD − 4.68 days, 95% CI − 6.22 to −3.14), although heterogeneity was very high (I^2^ = 98.74%; Figure 3A). When stratified by intervention type, the pooled effect remained significant in the prebiotic subgroup (MD − 5.32 days, 95% CI − 8.78 to −1.86) and the probiotic subgroup (MD − 4.46 days, 95% CI − 6.18 to −2.74), while the synbiotic subgroup was represented by a single study (MD − 7.83 days, 95% CI − 13.62 to −2.04). There was no significant difference between intervention subgroups (Qb = 1.29, *p* = 0.53).

In the sensitivity analysis, the overall reduction in hospital stay remained statistically significant (MD − 3.90 days, 95% CI − 5.93 to −1.87; Figure 3B), with persistent but lower heterogeneity than in the full analysis (I^2^ = 90.30%). The pooled effect remained significant in the prebiotic subgroup (MD − 5.32 days, 95% CI − 8.78 to −1.86) and the probiotic subgroup (MD − 3.13 days, 95% CI − 5.66 to −0.60), whereas the synbiotic subgroup again consisted of a single study (MD − 7.83 days, 95% CI − 13.62 to −2.04). Subgroup differences remained non-significant (Qb = 2.58, *p* = 0.27). When hospital stay was stratified by study design, both non-randomized studies (MD −5.27 days, 95% CI −8.90 to −1.64) and randomized controlled trials (MD −4.54 days, 95% CI −6.31 to −2.77) showed reductions in hospital stay, with no evidence of a subgroup difference (Qb = 0.13, *p* = 0.72; Supplementary Figure S1A).

#### Mortality

**Figure 4:**
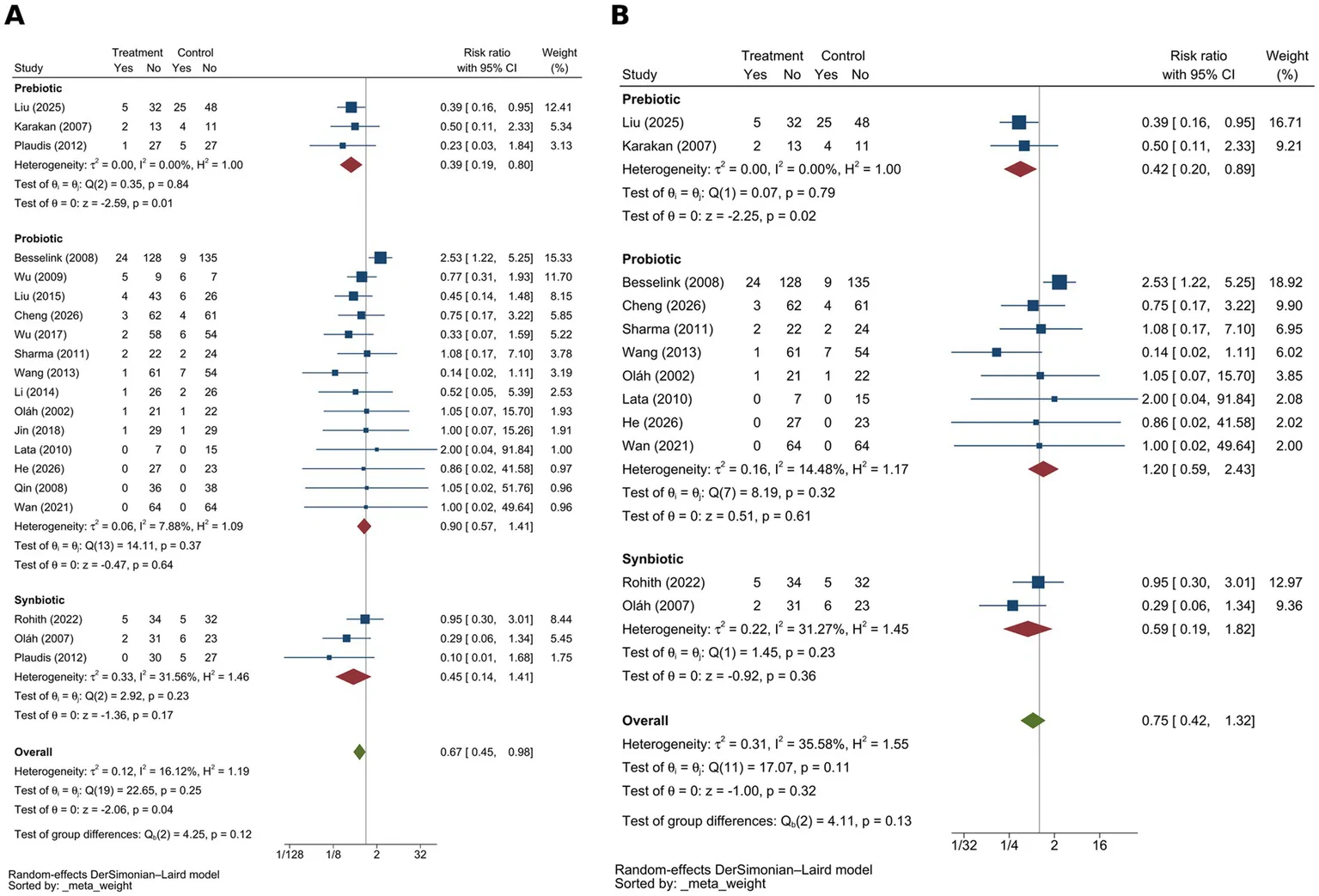
Effect of pre-, pro-, and synbiotics on mortality in acute pancreatitis. **(A)** Forest plot of the full-analysis set, subgrouped by intervention type (prebiotic, probiotic, and synbiotic). **(B)** Forest plot of the sensitivity-analysis set, subgrouped by intervention type. Risk ratios below 1 favor microbiota-directed interventions. Besselink et al. (36) should not be regarded simply as a statistical outlier; rather, it was a large, well-conducted multicenter RCT whose effect estimate was in the opposite direction to that observed in most of the smaller studies.

In the full-analysis set, microbiota-directed interventions were associated with a statistically significant reduction in mortality (RR 0.67, 95% CI 0.45 to 0.98; Figure 4A), with low overall heterogeneity (I^2^ = 16.12%). The prebiotic subgroup showed a significant reduction in mortality (RR 0.39, 95% CI 0.19 to 0.80), whereas the probiotic subgroup (RR 0.90, 95% CI 0.57 to 1.41) and synbiotic subgroup (RR 0.45, 95% CI 0.14 to 1.41) did not reach statistical significance. There was no significant subgroup difference by intervention type (Qb = 4.25, *p* = 0.12).

In the sensitivity analysis, the pooled effect on mortality was attenuated and no longer statistically significant (RR 0.75, 95% CI 0.42 to 1.32; Figure 4B), with moderate heterogeneity (I^2^ = 35.58%). The prebiotic subgroup remained statistically significant (RR 0.42, 95% CI 0.20 to 0.89), whereas the probiotic subgroup (RR 1.20, 95% CI 0.59 to 2.43) and synbiotic subgroup (RR 0.59, 95% CI 0.19 to 1.82) did not. Subgroup differences were not statistically significant (Qb = 4.11, *p* = 0.13).

When mortality was stratified by study design, the pooled effect was statistically significant among non-randomized studies (RR 0.39, 95% CI 0.21 to 0.72) but not among randomized controlled trials (RR 0.79, 95% CI 0.46 to 1.34), although the difference between study designs did not reach statistical significance (Qb = 2.85, *p* = 0.09; Supplementary Figure S1B).

#### Subgroup analysis

Additional exploratory subgroup analyses were performed to evaluate whether the hospital-stay effect could be explained by clinical or intervention-related sources of heterogeneity. When stratified by disease severity, the reduction in hospital stay was larger among studies enrolling severe acute pancreatitis patients (MD −4.94 days, 95% CI −6.66 to −3.22), whereas the mild-to-moderate subgroup was represented by a single study (MD −0.66 days, 95% CI −0.72 to −0.60), and one study had unspecified severity (MD −3.54 days, 95% CI −11.72 to 4.64; Supplementary Figure S6). Subgroup differences by disease severity were statistically significant, although residual heterogeneity remained high within the severe subgroup. When stratified by timing of intervention initiation, the pooled estimates were MD −6.32 days (95% CI −7.27 to −5.37) for interventions initiated within 48 h, MD −3.36 days (95% CI −12.76 to 6.04) for interventions initiated within 72 h, and MD −4.19 days (95% CI −5.97 to −2.41) for studies in which timing was unclear or not sufficiently specified; subgroup differences were not statistically significant (Supplementary Figure S7). When stratified by intervention duration, hospital stay was reduced in the ≤7-day subgroup (MD −7.13 days, 95% CI −9.11 to −5.15), the 8–14-day subgroup (MD −4.07 days, 95% CI −6.58 to −1.55), and studies with unspecified duration (MD −4.83 days, 95% CI −8.03 to −1.63), whereas the 15–28-day subgroup did not show a significant reduction (MD 2.85 days, 95% CI −4.90 to 10.61; Supplementary Figure S8). Comparator type also appeared to contribute to heterogeneity. The pooled effect was significant in studies using standard care controls (MD −8.76 days, 95% CI −12.47 to −5.05), standard enteral nutrition controls (MD −3.82 days, 95% CI −5.82 to −1.83), and other controls (MD −7.15 days, 95% CI −9.18 to −5.12), but not in placebo-controlled studies (MD −2.00 days, 95% CI −6.54 to 2.55; Supplementary Figure S9). Subgrouping by formulation category showed significant reductions for capsule/tablet formulations (MD −4.58 days, 95% CI −6.83 to −2.32) and unclear formulations (MD −5.37 days, 95% CI −6.77 to −3.96), while powder formulations did not show a significant pooled effect (MD −2.47 days, 95% CI −8.46 to 3.52; Supplementary Figure S10). Additional genus-level exploratory analyses were performed according to the reported presence of Lactobacillus, Bifidobacterium, Bacillus, Enterococcus, Lactococcus, and Streptococcus in the administered formulation. The subgroup analyses also allowed us to identify candidate intervention signals. For hospital stay, formulations containing Bifidobacterium, Lactobacillus, and Enterococcus showed favorable exploratory pooled estimates, suggesting that these genera may merit further evaluation in future trials. However, these findings should be interpreted cautiously because most interventions were multi-component preparations, strain-level reporting was incomplete, and genus-level classification cannot isolate the independent effect of a specific strain. In contrast, subgrouping by single- versus multi-strain formulation did not show a clear subgroup difference, indicating that the available evidence does not support a simple conclusion that multi-strain preparations are superior to single-strain preparations (Supplementary Figures S11–S17).

Additional exploratory subgroup analyses were also performed for mortality. Overall, these analyses did not identify a consistent mortality benefit across clinically defined strata. Mortality did not differ significantly by disease severity (subgroup difference *p* = 0.96) or timing of intervention initiation (subgroup difference *p* = 0.69). In contrast, subgroup differences were observed according to intervention duration (*p* = 0.01), comparator category (*p* = 0.05), and formulation category (*p* = 0.01). The 15–28-day duration subgroup showed an increased mortality risk (RR 2.43, 95% CI 1.19 to 5.00), whereas shorter or unspecified durations did not show a clear significant effect. By comparator category, studies using standard enteral nutrition as the control showed a favorable mortality signal (RR 0.38, 95% CI 0.15 to 0.95), while placebo-controlled studies showed a non-significant trend toward increased mortality (RR 1.78, 95% CI 0.99 to 3.21). By formulation, capsule/tablet preparations were associated with lower mortality (RR 0.47, 95% CI 0.24 to 0.90), whereas powder formulations were associated with increased mortality (RR 2.16, 95% CI 1.11 to 4.18). Subgrouping by single- versus multi-strain formulation did not show a significant subgroup difference (*p* = 0.19). Genus-level analyses did not support a robust mortality benefit for Lactobacillus- or Bifidobacterium-containing formulations, while Bacillus-containing formulations showed a favorable signal (RR 0.28, 95% CI 0.08 to 0.91) with only borderline between-subgroup evidence (*p* = 0.06). Enterococcus-containing formulations also showed a favorable but non-significant signal (RR 0.46, 95% CI 0.20 to 1.04; subgroup difference *p* = 0.08). In contrast, Lactococcus-containing formulations were associated with increased mortality (RR 2.51, 95% CI 1.22 to 5.14; subgroup difference *p* < 0.01), largely reflecting the contribution of the Besselink et al. study. These findings suggest that mortality effects are formulation- and context-dependent, but remain insufficient for definitive strain-specific recommendations (Supplementary Figures S12–S32).

### Secondary outcomes

#### Infection-related outcomes

**Figure 5:**
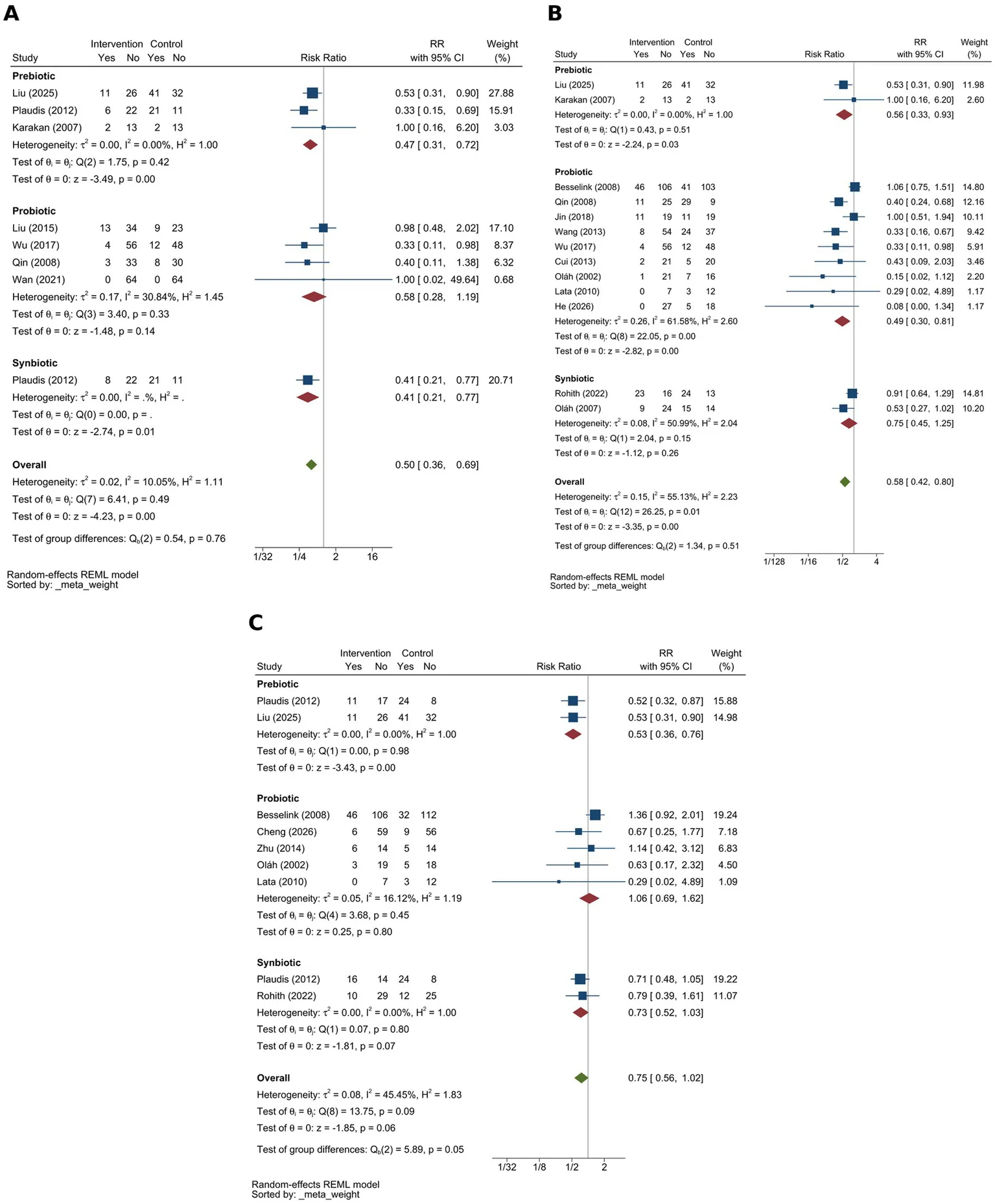
Effect of pre-, pro-, and synbiotics on infection-related outcomes in acute pancreatitis. **(A)** Total infection. **(B)** Infectious morbidity. **(C)** Infected pancreatic necrosis. All analyses are subgrouped by intervention type.

Microbiota-directed interventions were associated with a significant reduction in total infection in the full-analysis set (RR 0.50, 95% CI 0.36 to 0.69; Figure 5A), with low heterogeneity (I^2^ = 10.05%). The pooled effect remained significant after sensitivity exclusion (RR 0.56, 95% CI 0.34 to 0.94; Supplementary Figure S2A).

For infectious morbidity, the full-analysis set showed a significant reduction with microbiota-directed interventions (RR 0.58, 95% CI 0.42 to 0.80; Figure 5B), although heterogeneity was moderate (I^2^ = 55.13%). This finding remained significant in the sensitivity analysis (RR 0.60, 95% CI 0.41 to 0.87; Supplementary Figure S2B).

By contrast, infected pancreatic necrosis was not significantly reduced in the full-analysis set (RR 0.75, 95% CI 0.56 to 1.02; Figure 5C) or in the sensitivity analysis (RR 0.82, 95% CI 0.47 to 1.44; Supplementary Figure S2C).

#### Organ failure, surgery, and SIRS

**Figure 6:**
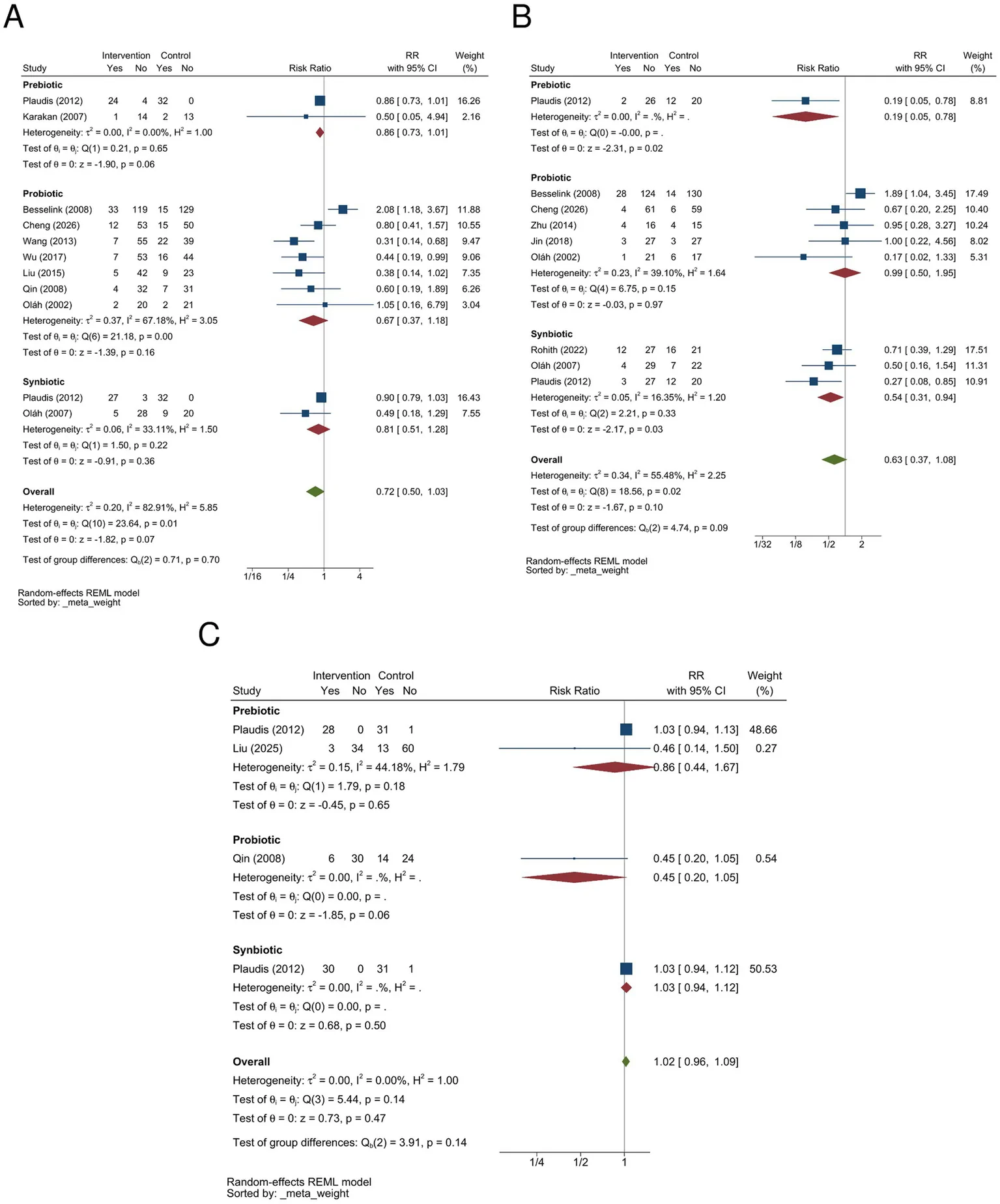
Effect of pre-, pro-, and synbiotics on systemic inflammatory response and procedure-related outcomes in acute pancreatitis. **(A)** Organ failure or multiple organ failure. **(B)** Need for operation. **(C)** Systemic inflammatory response syndrome. All analyses are subgrouped by intervention type.

Microbiota-directed interventions were not associated with a statistically significant reduction in organ failure or multiple organ failure in the full-analysis set (RR 0.72, 95% CI 0.50 to 1.03; Figure 6A) or in the sensitivity analysis (RR 0.74, 95% CI 0.38 to 1.49; Supplementary Figure S3A).

Similarly, the need for operation was not significantly different between intervention and control groups in either the full analysis (RR 0.72, 95% CI 0.50 to 1.03; Figure 6B) or the sensitivity analysis (RR 0.63, 95% CI 0.37 to 1.08; Supplementary Figure S3B).

No significant effect was observed for systemic inflammatory response syndrome in the full-analysis set (RR 1.02, 95% CI 0.96 to 1.09; Figure 6C). After sensitivity exclusion, only a single prebiotic study remained available for this outcome (RR 0.46, 95% CI 0.14 to 1.50; Supplementary Figure S3C).

#### Small-study effects and influence analyses

Visual inspection of funnel plots for the primary outcomes is shown in Supplementary Figure S4, and formal small-study-effect analyses are summarized in Supplementary Table 5. For mortality, neither Egger’s nor Begg’s test suggested significant small-study effects in the full or sensitivity analyses. In the full-analysis set, trim-and-fill imputed one potentially missing study; the observed pooled effect on the log risk ratio scale was −0.453 (95% CI −0.892 to −0.015), and the adjusted estimate was attenuated to −0.412 (95% CI −0.845 to 0.021). In the sensitivity analysis, no studies were imputed.

For hospital stay, Egger’s test was not significant in either the full or sensitivity analyses, whereas Begg’s test was significant only in the full-analysis set. Trim-and-fill suggested potential missing studies for hospital stay in both the full-analysis and sensitivity-analysis sets, but these models produced convergence warnings; therefore, these results should be interpreted cautiously (Supplementary Figure S4; Supplementary Table 5).

Leave-one-out analyses are presented in Supplementary Figure S5. For hospital stay, the pooled effect remained statistically significant after sequential omission of each study-level comparison in both the full-analysis and sensitivity-analysis sets, indicating that the overall finding was not driven by any single comparison. In contrast, the mortality findings were more sensitive to individual exclusions. In the full-analysis set, omission of Besselink et al. (36) shifted the pooled estimate to a statistically significant reduction in mortality.

A dedicated sensitivity analysis was performed to evaluate the influence of the Besselink et al. trial. After excluding this study, the pooled reduction in hospital stay remained statistically significant (MD −4.93 days, 95% CI −6.43 to −3.43), with persistent substantial heterogeneity (I^2^ = 98.70%). In intervention-category subgroups, the pooled estimates remained favorable for prebiotics (MD −5.32 days, 95% CI −8.78 to −1.86), probiotics (MD −4.76 days, 95% CI −6.43 to −3.10), and synbiotics (MD −7.83 days, 95% CI −13.62 to −2.04), with no significant subgroup difference (Supplementary Figure S33). For mortality, exclusion of Besselink et al. materially changed the pooled estimate. The overall pooled effect shifted toward a significant reduction in mortality (RR 0.53, 95% CI 0.36 to 0.76), with no residual statistical heterogeneity (I^2^ = 0.00%). In subgroup analyses, the prebiotic subgroup remained statistically significant (RR 0.39, 95% CI 0.19 to 0.80), whereas the probiotic subgroup showed a borderline favorable estimate (RR 0.61, 95% CI 0.37 to 1.01), and the synbiotic subgroup remained non-significant (RR 0.45, 95% CI 0.14 to 1.42). These findings indicate that Besselink et al. had a major influence on the mortality estimate (Supplementary Figure S34).

#### Safety outcomes and adverse-events

Study-level safety and adverse-event reporting was heterogeneous across the included studies (Supplementary Table S6). Many studies did not separately report intervention-related adverse events, including bacteremia, fungemia, bowel or intestinal ischemia, gastrointestinal intolerance, diarrhea, vomiting, or probiotic-related infection. Therefore, absence of reporting was not interpreted as absence of events. The most prominent safety signal was bowel or intestinal ischemia. In the Besselink et al. trial, bowel ischemia occurred in 9/152 patients receiving probiotics compared with 0/144 patients receiving placebo. Zhu et al. also reported intestinal ischemia or necrosis in 2/20 patients in the probiotic group compared with 0/19 patients in the control group. These findings were considered clinically important safety signals, particularly because Besselink et al. was the largest multicenter RCT and also showed increased mortality in the probiotic arm. Bacteremia or fungemia was rarely reported as a separate safety endpoint. Rohith et al. reported bacteremia in 5/39 patients in the synbiotic group and 13/37 patients in the control group, while He et al. mentioned fungemia as an endpoint but did not provide separately extractable event counts. Wan et al. explicitly reported no probiotic-related serious adverse events in either group. Minor gastrointestinal symptoms were inconsistently reported. Wan et al. reported abdominal discomfort in 12/64 probiotic-treated patients versus 9/64 controls, with nausea, abdominal pain, and flatulence also reported in the probiotic arm. Dou et al. reported diarrhea in 1/82 versus 1/78 patients and vomiting/nausea in 2/82 versus 1/78 patients, with total adverse reactions in 4/82 versus 2/78 patients.

Several studies reported aggregate adverse reactions or pancreatitis-related complications rather than clearly intervention-attributable adverse events. Wang et al. reported no adverse reactions in either group, Zhao et al. reported total adverse reactions in 0/34 intervention patients versus 2/32 controls, Fang et al. reported total adverse reactions in 5/34 versus 3/34 patients, and Ma et al. reported total complications in 7/30 versus 11/30 patients. Other reported complications included peripancreatic infection, upper gastrointestinal bleeding, renal failure, pancreatic pseudocyst, pancreatic abscess, pancreatic calcification, pneumonia, and urinary infection. Overall, the safety synthesis indicates that minor adverse events were not consistently increased across studies, but reporting was incomplete, and serious safety concerns remain driven mainly by the intestinal ischemia and mortality signal observed in the Besselink et al. trial.

## Discussion

This systematic review and meta-analysis evaluated the safety and efficacy of prebiotics, probiotics, and synbiotics in patients with acute pancreatitis using evidence from randomized and observational studies. The findings indicate that microbiota-based interventions may provide modest clinical benefits, particularly in reducing length of hospital stay, while their effects on major clinical outcomes such as mortality, infection, and organ failure were less consistent. The apparent reduction in hospital stay despite no consistent reduction in infected pancreatic necrosis, organ failure, or SIRS requires cautious interpretation. Hospital stay is a complex recovery-related and healthcare-utilization endpoint rather than a direct measure of severe disease modification. Severe complications such as infected pancreatic necrosis and multiple organ failure are relatively infrequent binary outcomes and may be underpowered in the available trials, whereas length of stay may be more sensitive to smaller differences in clinical recovery. Microbiota-directed interventions may influence earlier recovery milestones, such as tolerance of enteral nutrition, gastrointestinal motility, abdominal distension, pain control, intestinal barrier function, or reduced need for prolonged nutritional support, without necessarily preventing established necrosis, organ failure, or systemic inflammatory response. In addition, discharge timing is influenced by local practice patterns, nutritional protocols, comparator care, healthcare-system factors, and non-standardized discharge criteria. Therefore, the hospital-stay signal should not be interpreted as proof that these interventions reduce severe pancreatitis-related complications. Rather, it should be viewed as a recovery signal that requires confirmation in standardized, adequately powered trials with harmonized discharge criteria and robust assessment of safety-critical outcomes.

In addition, the mortality signal observed in the full analysis was attenuated after sensitivity analysis and appeared more dependent on study design and individual influential studies. Although the prebiotic subgroup showed a favorable estimate for hospital stay, this result should be interpreted cautiously because the subgroup included a small number of studies and participants. The apparently robust effect may therefore be susceptible to small-study effects, baseline imbalance, selective reporting, or center-specific differences in clinical and nutritional care. Accordingly, this finding was considered exploratory and hypothesis-generating rather than confirmatory. The type of prebiotic substrate and the timing of nutritional initiation are likely important modifiers of treatment effect, but the current evidence does not allow these factors to be disentangled.

Reduction in length of hospital stay was the most consistent beneficial outcome observed in this analysis. This finding is clinically important because prolonged hospitalization in acute pancreatitis is associated with increased complications, healthcare burden, and delayed recovery. Several individual trials reported improvements in inflammatory markers, intestinal barrier function, immune indices, or clinical recovery when probiotics were combined with early enteral nutrition, pharmacological therapy, microecological enteral nutrition, or adjunctive herbal therapy (1, 8–10). The biological plausibility of this effect is supported by evidence linking restoration of intestinal barrier integrity and reduction of systemic inflammation with improved clinical outcomes.

In contrast, the present analysis did not demonstrate robust and consistent benefits for mortality, organ failure, infected pancreatic necrosis, or need for operation. This is clinically important because severe AP is driven by multiple interacting mechanisms, including pancreatic necrosis, systemic inflammation, organ dysfunction, comorbidities, timing of supportive care, and infection control. The absence of strong effects on these outcomes likely reflects the multifactorial pathophysiology of severe acute pancreatitis, in which inflammatory response, pancreatic necrosis, and comorbid conditions contribute substantially to clinical progression.

These findings are broadly consistent with previous systematic reviews, which reported possible improvements in some recovery-related outcomes but limited or uncertain effects on mortality and organ failure (2, 3, 5, 6). They are also consistent with clinical uncertainty raised by studies in predicted severe AP, including reports showing no clear positive or negative effect of probiotic prophylaxis on infectious complications or mortality (11, 36). Therefore, the apparent benefit on hospital stay should be interpreted alongside the weaker and less stable evidence for major safety and efficacy endpoints.

Considerable heterogeneity across studies represents an important factor influencing the interpretation of these findings. Included studies differed in disease severity, timing of intervention, probiotic strains, single- versus multi-strain formulations, prebiotic components, synbiotic combinations, dose, duration of therapy, co-interventions, and comparator care. Variability in disease severity and supportive care protocols may further explain inconsistent treatment effects. Outcome definitions were also inconsistent, particularly for infection-related outcomes and organ failure. These differences likely contributed to the high heterogeneity observed for hospital stay and limit the certainty with which pooled estimates can be generalized to routine clinical practice. Such methodological heterogeneity has been repeatedly identified as a major limitation in previous meta-analyses evaluating microbiota-based interventions in acute pancreatitis (5, 6).

The additional subgroup analyses in our work provide a more clinically nuanced interpretation of the pooled findings. For hospital stay, the magnitude of benefit varied across clinically meaningful strata, with stronger reductions observed in studies enrolling severe acute pancreatitis patients, shorter intervention-duration categories, non-placebo comparator settings, and capsule/tablet formulations. Genus-level analyses also suggested exploratory favorable recovery-related signals for formulations containing Bifidobacterium, Lactobacillus, and Enterococcus. However, these findings should be interpreted as candidate signals, because most interventions were multi-component formulations, strain-level reporting was incomplete, and the independent contribution of each genus could not be isolated. In contrast, the mortality subgroup analyses were less consistent and appeared more context-dependent. Although favorable signals were observed in some subgroups, including capsule/tablet formulations, standard-enteral-nutrition comparator studies, and Bacillus- or Enterococcus-containing formulations, the increased mortality signals observed in powder and Lactococcus-containing formulations were largely influenced by the Besselink et al. trial, which remains a key safety counter-signal for multispecies live probiotic prophylaxis in predicted severe acute pancreatitis. Therefore, the subgroup findings improve interpretation of heterogeneity and help identify formulations that may merit future study, but they do not justify definitive recommendations for any specific strain, substrate, formulation, or timing strategy at this stage.

Safety remains a critical consideration when evaluating probiotic therapy in patients with severe acute pancreatitis. Although most studies reported acceptable safety profiles, concerns have been raised regarding potential adverse outcomes in critically ill patients receiving probiotic supplementation. The divergent findings across trials and the influence of large studies on mortality estimates emphasize the need for careful patient selection, standardized formulations, predefined safety monitoring, and transparent adverse-event reporting in future trials.

The Besselink et al. trial requires special consideration because it was one of the methodologically strongest studies included in this review, while also showing a clear safety signal in the opposite direction to many smaller trials. Excluding this study shifted the pooled mortality estimate toward an apparent benefit; however, this should not be interpreted as confirmation of probiotic safety. Rather, it demonstrates that the overall mortality conclusion is highly dependent on a single large, high-quality trial conducted in a particularly vulnerable population of patients with predicted severe acute pancreatitis. Several factors may explain why this trial diverged from smaller studies. Besselink et al. enrolled patients with predicted severe disease, administered a multispecies live probiotic preparation early in the disease course, and continued treatment for a prolonged period. In severe acute pancreatitis, intestinal barrier failure, ileus, systemic inflammation, increased intra-abdominal pressure, microcirculatory dysfunction, and splanchnic hypoperfusion may predispose patients to bacterial translocation and non-occlusive mesenteric ischemia. Under these conditions, enteral administration of live microorganisms may carry risks that are not observed in mild disease, in more stable patients, or with non-live prebiotic substrates. Potential mechanisms include impaired gut barrier integrity, increased local metabolic demand, gas production, mucosal stress, bacterial translocation, and exacerbation of intestinal ischemia. The excess bowel ischemia and mortality reported in Besselink et al. therefore represent a biologically and clinically important safety signal.

These findings highlight the need to distinguish recovery-related outcomes from safety-critical outcomes and to avoid treating all microbiota-directed interventions as interchangeable. Although several smaller studies suggest possible reductions in hospital stay and some infection-related outcomes, the Besselink et al. trial prevents any conclusion that live probiotic prophylaxis is uniformly safe in predicted severe acute pancreatitis. This distinction is particularly important because prebiotics are non-living substrates intended to modify the intestinal microbiome or support beneficial microbial metabolism, whereas probiotics and synbiotics contain live microorganisms. Therefore, live probiotic preparations may carry risks that are not shared by prebiotic substrates, especially in critically ill patients with shock, ileus, impaired mesenteric perfusion, or high risk of bowel ischemia. Notably, mortality subgroup analyses showed adverse signals for powder formulations and Lactococcus-containing formulations, but both signals were largely driven by the Besselink et al. trial and should be interpreted as safety signals requiring further investigation rather than as evidence of independent harm from these categories. Overall, the current evidence does not support routine use of multispecies live probiotics in critically ill or predicted severe acute pancreatitis patients. Future trials should evaluate live probiotic, prebiotic, and synbiotic strategies separately; stratify by disease severity and hemodynamic risk; and prospectively capture adverse events such as bowel ischemia, bacteremia, fungemia, sepsis, organ failure, and mortality.

Exploratory genus-level subgroup analyses provided additional insight into which formulations may deserve further investigation. For hospital stay, formulations containing Bifidobacterium, Lactobacillus, and Enterococcus showed the most favorable recovery-related signals. These genera may plausibly influence gut barrier function, intestinal dysbiosis, feeding tolerance, and immune modulation, although the present analysis cannot isolate their independent effects because most preparations were multi-component products. For mortality, the signal was less consistent. Bacillus-containing formulations showed a favorable estimate, and Enterococcus-containing formulations showed a favorable but non-significant trend, whereas Lactobacillus- and Bifidobacterium-containing formulations did not demonstrate a robust mortality benefit. Conversely, Lactococcus-containing formulations were associated with an adverse mortality signal, largely driven by the Besselink et al. trial. Therefore, the available evidence suggests that potential benefit and harm are formulation-specific and context-dependent. These findings support further evaluation of clearly defined Bifidobacterium-, Lactobacillus-, Enterococcus-, Bacillus-based strategies in future clinical trials.

The main strength of the present review is its broad evaluation of prebiotics, probiotics, and synbiotics across multiple clinically relevant endpoints, including hospital stay, mortality, infection-related outcomes, organ failure, need for operation, and SIRS. Unlike previous analyses that focused primarily on probiotic therapy alone, this study evaluated prebiotics, probiotics, and synbiotics and allows comparison across intervention categories. Nevertheless, several limitations should be acknowledged. The number of studies contributing data to specific outcomes varied, and substantial heterogeneity in study design and intervention protocols may limit the precision of pooled estimates. In addition, incomplete methodological reporting in some studies introduces potential risk of bias. These limitations highlight the need for large, well-designed randomized controlled trials using standardized treatment protocols, uniform outcome definitions, and adequate power to clarify the clinical effectiveness and safety of microbiota-based therapies in acute pancreatitis. A further limitation is that some continuous outcomes were originally reported as medians with ranges or interquartile ranges and required conversion to means and standard deviations. Although established conversion methods were used, these transformations may introduce uncertainty, particularly for skewed outcomes such as hospital stay, and may have contributed to residual heterogeneity.

## Conclusion

In this systematic review and meta-analysis, microbiota-directed adjunctive interventions were associated with shorter hospital stay in some analyses; however, this finding should be interpreted with substantial caution because of extreme clinical, methodological, and intervention-related heterogeneity. The hospital-stay signal was not consistently accompanied by reductions in infected pancreatic necrosis, organ failure, SIRS, mortality, or other safety-critical outcomes, and therefore should not be considered evidence of reduced disease severity or practice-changing clinical benefit. The apparent mortality signal was attenuated in sensitivity analysis and appeared dependent on study design and trial selection. Current evidence is insufficient to support routine or unrestricted clinical use of prebiotics, probiotics, or synbiotics in acute pancreatitis, especially in severe or critically ill patients. This caution is particularly important for live probiotic and synbiotic preparations, as live probiotics do not have homogeneous clinical safety across acute pancreatitis populations. The Besselink et al. trial remains a pivotal safety counter-signal against routine multispecies live probiotic prophylaxis in predicted severe acute pancreatitis, particularly in patients with shock, ileus, impaired gut barrier function, splanchnic hypoperfusion, or risk of bowel ischemia, in whom live microbial administration may carry clinically important risks, including microbial translocation, sepsis, and non-occlusive intestinal ischemia. Prebiotic interventions may represent a biologically distinct and potentially safer strategy because they do not introduce live microorganisms; however, the prebiotic evidence base remains small and should be considered hypothesis-generating for future investigations. Future trials should evaluate clearly defined prebiotic substrates, probiotic strains, or synbiotic formulations separately. Such trials should be adequately powered, multicenter, severity-stratified, and formulation-specific, with standardized timing, dose, duration, nutritional co-interventions, discharge criteria, harmonized outcome definitions, and rigorous prospective monitoring of safety-critical outcomes including bowel ischemia, bacteremia, fungemia, sepsis, organ failure, and mortality.

## Data Availability

The original contributions presented in the study are included in the article/Supplementary material, further inquiries can be directed to the corresponding author.
