# Supplementary material for: Safety and efficacy of microbiota-directed adjunctive therapy in acute pancreatitis: a systematic review and meta-analysis of prebiotics, probiotics, and synbiotics
Source: Front Med (Lausanne). 2026 Sep 11;13:1873327. doi: 10.3389/fmed.2026.1873327 (PMC13612259; doi:10.3389/fmed.2026.1873327)
Supplement: Supplementary file 1 [file Image_1.DOCX]

**Figure S1.** Primary outcomes stratified by study design. (A) Hospital stay subgrouped by randomized controlled trials and non-randomized studies. (B) Mortality subgrouped by randomized controlled trials and non-randomized studies.

**Figure S2.** Sensitivity analyses of infection-related outcomes. (A) Total infection. (B) Infectious morbidity. (C) Infected pancreatic necrosis. All analyses are subgrouped by intervention type after sensitivity exclusion.

**Figure S3.** Sensitivity analyses of systemic inflammatory response and procedure-related outcomes. (A) Organ failure or multiple organ failure. (B) Need for operation. (C) Systemic inflammatory response syndrome. All analyses are subgrouped by intervention type after sensitivity exclusion.

**Figure S4.** Funnel plots for the primary outcomes. (A) Hospital stay in the full-analysis set. (B) Hospital stay in the sensitivity-analysis set. (C) Mortality in the full-analysis set. (D) Mortality in the sensitivity-analysis set.

**Figure S5.** Leave-one-out analyses for the primary outcomes. (A) Hospital stay in the full-analysis set. (B) Hospital stay in the sensitivity-analysis set. (C) Mortality in the full-analysis set. (D) Mortality in the sensitivity-analysis set.

**Figure S6.** Subgroup analysis of hospital stay according to disease severity.

**Figure S7.** Subgroup analysis of hospital stay according to the timing of intervention initiation.

**Figure S8.** Subgroup analysis of hospital stay according to intervention duration.

**Figure S9.** Subgroup analysis of hospital stay according to control category.

**Figure S10.** Subgroup analysis of hospital stay according to formulation category.

**Figure S11.** Subgroup analysis of hospital stay according to single-strain versus multi-strain formulation.

**Figure S12.** Subgroup analysis of hospital stay according to the presence of Lactobacillus in the administered formulation.

**Figure S13.** Subgroup analysis of hospital stay according to the presence of Bifidobacterium in the administered formulation.

**Figure S14.** Subgroup analysis of hospital stay according to the presence of Bacillus in the administered formulation.

**Figure S15.** Subgroup analysis of hospital stay according to the presence of Enterococcus in the administered formulation.

**Figure S16.** Subgroup analysis of hospital stay according to the presence of Lactococcus in the administered formulation.

**Figure S17.** Subgroup analysis of hospital stay according to the presence of Streptococcus in the administered formulation.

**Figure S18.** Additional sensitivity analysis of hospital stay after exclusion of Besselink et al. (2008).

**Figure S19.** Additional sensitivity analysis of hospital stay after exclusion of Wan et al. (2021).

**Figure S20.** Additional sensitivity analysis of hospital stay after simultaneous exclusion of Besselink et al. (2008) and Wan et al. (2021).

**Figure S21.** Subgroup analysis of mortality according to disease severity.

**Figure S22.** Subgroup analysis of mortality according to the timing of intervention initiation.

**Figure S23.** Subgroup analysis of mortality according to intervention duration.

**Figure S24.** Subgroup analysis of mortality according to control category.

**Figure S25.** Subgroup analysis of mortality according to formulation category.

**Figure S26.** Subgroup analysis of mortality according to single-strain versus multi-strain formulation.

**Figure S27.** Subgroup analysis of mortality according to the presence of Lactobacillus in the administered formulation.

**Figure S28.** Subgroup analysis of mortality according to the presence of Bifidobacterium in the administered formulation.

**Figure S29.** Subgroup analysis of mortality according to the presence of Bacillus in the administered formulation.

**Figure S30.** Subgroup analysis of mortality according to the presence of Enterococcus in the administered formulation.

**Figure S31.** Subgroup analysis of mortality according to the presence of Lactococcus in the administered formulation.

**Figure S32.** Subgroup analysis of mortality according to the presence of Streptococcus in the administered formulation.

**Figure S33.** Sensitivity analysis of hospital stay excluding the Besselink et al study

**Figure S34.** Sensitivity analysis of mortality excluding the Besselink et al study
