# Supplementary material for: Safety and efficacy of microbiota-directed adjunctive therapy in acute pancreatitis: a systematic review and meta-analysis of prebiotics, probiotics, and synbiotics
Source: Front Med (Lausanne). 2026 Sep 11;13:1873327. doi: 10.3389/fmed.2026.1873327 (PMC13612259; doi:10.3389/fmed.2026.1873327)
Supplement: Supplementary file 2 [file Table_1.DOCX]

**Table S1- Keywords used for search in different databases**

| **Keywords for pre-, pro and syn-biotics** | **Keywords for pancreatitis** |
| --- | --- |
| Prebiotic*  Probiotic*  Symbiotic*  Synbiotic*  microbiota | Pancreatitis  Acute Pancreatitis  Acute Edematous Pancreat*  Peripancreatic Fat Necros*  Pancreatic Parenchymal Edema  Pancreatic Parenchyma with Edema  Severe Acute Pancreatitis |
