# Supplementary material for: Safety and efficacy of microbiota-directed adjunctive therapy in acute pancreatitis: a systematic review and meta-analysis of prebiotics, probiotics, and synbiotics
Source: Front Med (Lausanne). 2026 Sep 11;13:1873327. doi: 10.3389/fmed.2026.1873327 (PMC13612259; doi:10.3389/fmed.2026.1873327)
Supplement: Supplementary file 3 [file Table_2.DOCX]

**Table S2- Search strategy and the associated results in different databases**

| **Database** | **Search strategy** | **Results** |
| --- | --- | --- |
| **PubMed** | #1: ((((Prebiotic*[Title/Abstract]) OR (Probiotic*[Title/Abstract])) OR (Symbiotic*[Title/Abstract])) OR (Synbiotic*[Title/Abstract])) OR (microbiota[Title/Abstract]) | [207,751](https://pubmed.ncbi.nlm.nih.gov/?term=%28%28%28%28Prebiotic%2A%5BTitle%2FAbstract%5D%29+OR+%28Probiotic%2A%5BTitle%2FAbstract%5D%29%29+OR+%28Symbiotic%2A%5BTitle%2FAbstract%5D%29%29+OR+%28Synbiotic%2A%5BTitle%2FAbstract%5D%29%29+OR+%28microbiota%5BTitle%2FAbstract%5D%29&sort=) |
|  | #2: ((((((Pancreatitis[Title/Abstract]) OR (Acute Pancreatitis[Title/Abstract])) OR (Acute Edematous Pancreat*[Title/Abstract])) OR (Peripancreatic Fat Necros*[Title/Abstract])) OR (Pancreatic Parenchymal Edema[Title/Abstract])) OR (Pancreatic Parenchyma with Edema[Title/Abstract])) OR (Severe Acute Pancreatitis[Title/Abstract]) | [75,542](https://pubmed.ncbi.nlm.nih.gov/?term=%28%28%28%28%28%28Pancreatitis%5BTitle%2FAbstract%5D%29+OR+%28Acute+Pancreatitis%5BTitle%2FAbstract%5D%29%29+OR+%28Acute+Edematous+Pancreat%2A%5BTitle%2FAbstract%5D%29%29+OR+%28Peripancreatic+Fat+Necros%2A%5BTitle%2FAbstract%5D%29%29+OR+%28Pancreatic+Parenchymal+Edema%5BTitle%2FAbstract%5D%29%29+OR+%28Pancreatic+Parenchyma+with+Edema%5BTitle%2FAbstract%5D%29%29+OR+%28Severe+Acute+Pancreatitis%5BTitle%2FAbstract%5D%29&sort=) |
|  | #1 AND #2 | [509](https://pubmed.ncbi.nlm.nih.gov/?term=%28%28%28%28%28Prebiotic%2A%5BTitle%2FAbstract%5D%29+OR+%28Probiotic%2A%5BTitle%2FAbstract%5D%29%29+OR+%28Symbiotic%2A%5BTitle%2FAbstract%5D%29%29+OR+%28Synbiotic%2A%5BTitle%2FAbstract%5D%29%29+OR+%28microbiota%5BTitle%2FAbstract%5D%29%29+AND+%28%28%28%28%28%28%28Pancreatitis%5BTitle%2FAbstract%5D%29+OR+%28Acute+Pancreatitis%5BTitle%2FAbstract%5D%29%29+OR+%28Acute+Edematous+Pancreat%2A%5BTitle%2FAbstract%5D%29%29+OR+%28Peripancreatic+Fat+Necros%2A%5BTitle%2FAbstract%5D%29%29+OR+%28Pancreatic+Parenchymal+Edema%5BTitle%2FAbstract%5D%29%29+OR+%28Pancreatic+Parenchyma+with+Edema%5BTitle%2FAbstract%5D%29%29+OR+%28Severe+Acute+Pancreatitis%5BTitle%2FAbstract%5D%29%29&sort=) |
| **Scopus** | #3: ( TITLE-ABS-KEY ( Prebiotic* ) OR TITLE-ABS-KEY ( Probiotic* ) OR TITLE-ABS-KEY ( Symbiotic* ) OR TITLE-ABS-KEY ( Synbiotic* ) OR TITLE-ABS-KEY ( microbiota ) ) | 352,670 results |
|  | #4:  ( TITLE-ABS-KEY ( Pancreatitis ) OR TITLE-ABS-KEY ( Acute Pancreatitis ) OR TITLE-ABS-KEY ( Acute Edematous Pancreat* ) OR TITLE-ABS-KEY ( Peripancreatic Fat Necros* ) OR TITLE-ABS-KEY ( Pancreatic Parenchymal Edema ) OR TITLE-ABS-KEY ( Pancreatic Parenchyma with Edema ) OR TITLE-ABS-KEY ( Severe Acute Pancreatitis ) ) | 129,070 results |
|  | #3 AND #4 | 1,330 results |
| **Web of Science** | #5:  Prebiotic* (Topic) or Probiotic* (Topic) or Symbiotic* (Topic) or Synbiotic* (Topic) or microbiota (Topic) | [315,684](https://www.webofscience.com/wos/woscc/summary/90252c8f-34b6-4bfa-a780-2211ca457462-01adf3a1b3/relevance/1) |
|  | #6:  Pancreatitis (Topic) or Acute Pancreatitis (Topic) or Acute Edematous Pancreat* (Topic) or Peripancreatic Fat Necros* (Topic) or Pancreatic Parenchymal Edema (Topic) or Pancreatic Parenchyma with Edema (Topic) or Severe Acute Pancreatitis (Topic) | [81,694](https://www.webofscience.com/wos/woscc/summary/12c266c0-6d49-40ce-9be2-d45fb06142bc-01adf3ba06/relevance/1) |
|  | #5 AND #6 | [762](https://www.webofscience.com/wos/woscc/summary/b1d3f3ec-3d62-4e2b-b9a4-171720869765-01adf3c434/relevance/1) |
| **Embase** | #7  prebiotic*:ti,ab OR probiotic*:ti,ab OR symbiotic*:ti,ab OR synbiotic*:ti,ab OR microbiota:ti,ab | 246,101 |
|  | #8  pancreatitis:ti,ab OR "acute pancreatitis":ti,ab OR "acute edematous pancreat*":ti,ab OR "peripancreatic fat necros*":ti,ab OR "pancreatic parenchymal edema":ti,ab OR "pancreatic parenchyma with edema":ti,ab OR "severe acute pancreatitis":ti,ab | 123,855 |
|  | #7 AND #8 | 779 |
| **Cochrane** | #9  (prebiotic* OR probiotic* OR symbiotic* OR synbiotic* OR microbiota):ti,ab,kw | [22718](https://www.cochranelibrary.com/en/advanced-search?searchBy=-1&searchText=(prebiotic*+OR+probiotic*+OR+symbiotic*+OR+synbiotic*+OR+microbiota)&isWordVariations=&resultPerPage=25&searchType=advanced&forceTypeSelection=true&selectedType=central&displayText=&orderBy=relevancy&p_p_id=scolarissearchresultsportlet_WAR_scolarissearchresults&p_p_lifecycle=0&p_p_state=normal&p_p_mode=view&p_p_col_id=column-1&p_p_col_pos=1&p_p_col_count=2) |
|  | #10  (pancreatitis OR "acute pancreatitis" OR "acute edematous pancreat*" OR "peripancreatic fat necros*" OR "pancreatic parenchymal edema" OR "pancreatic parenchyma with edema" OR "severe acute pancreatitis"):ti,ab,kw | 6498 |
|  | #9 AND #10 | 122 |
