# Supplementary material for: Safety and efficacy of microbiota-directed adjunctive therapy in acute pancreatitis: a systematic review and meta-analysis of prebiotics, probiotics, and synbiotics
Source: Front Med (Lausanne). 2026 Sep 11;13:1873327. doi: 10.3389/fmed.2026.1873327 (PMC13612259; doi:10.3389/fmed.2026.1873327)
Supplement: Supplementary file 4 [file Table_3.DOCX]

**Supplementary Table 3. Risk of bias assessment of randomized controlled trials using the Cochrane Risk of Bias 2 tool.**

| **Study** | **Year** | **Randomization** | **Deviations** | **Missing data** | **Outcome measurement** | **Reported result** | **Overall** |
| --- | --- | --- | --- | --- | --- | --- | --- |
| Besselink | 2008 | Low | Low | Low | Low | Low | Low |
| Karakan | 2007 | Low | Low | Low | Low | Some concerns | Some concerns |
| Wan | 2021 | Low | Low | Low | Low | Low | Low |
| Rohith | 2022 | Low | Low | Low | Low | Low | Low |
| Sharma | 2011 | Some concerns | Low | Low | Low | Some concerns | Some concerns |
| Cui | 2013 | Some concerns | Some concerns | Low | Low | Some concerns | Some concerns |
| Wu | 2017 | Some concerns | High | Low | Some concerns | Some concerns | High |
| Cui | 2009 | High | Some concerns | Low | Low | Some concerns | High |
| Lata | 2010 | Low | Low | Low | Low | Some concerns | Some concerns |
| Li | 2014 | High | Some concerns | Low | Low | Some concerns | High |
| Qin | 2008 | High | Some concerns | Some concerns | Low | Some concerns | High |
| Oláh | 2007 | Low | Low | Low | Low | Some concerns | Some concerns |
| Oláh | 2002 | Low | Low | Low | Low | Some concerns | Some concerns |
| Wang | 2013 | Some concerns | Some concerns | Low | Low | Some concerns | Some concerns |
| Zhu | 2014 | Low | Low | Low | Low | Some concerns | Some concerns |
| Fang | 2018 | Some concerns | High | Low | High | Some concerns | High |
| Liao | 2020 | Some concerns | Some concerns | Low | Some concerns | Some concerns | Some concerns |
| Xiao | 2022 | Some concerns | High | Low | Some concerns | Some concerns | High |
| Zhao | 2024 | Some concerns | High | Low | High | Some concerns | High |
| He | 2026 | Low | Some concerns | Low | Low | Some concerns | Some concerns |
| Cheng | 2026 | Low | Low | Low | Low | Low | Low |
| Ma | 2025 | Low | Some concerns | Low | Some concerns | Some concerns | Some concerns |

RCT overall judgements use Low risk / Some concerns / High risk.
