# Supplementary material for: Safety and efficacy of microbiota-directed adjunctive therapy in acute pancreatitis: a systematic review and meta-analysis of prebiotics, probiotics, and synbiotics
Source: Front Med (Lausanne). 2026 Sep 11;13:1873327. doi: 10.3389/fmed.2026.1873327 (PMC13612259; doi:10.3389/fmed.2026.1873327)
Supplement: Supplementary file 5 [file Table_4.DOCX]

**Supplementary Table 4. Quality assessment of non-randomized studies using the Newcastle–Ottawa Scale.**

| **Study** | **Year** | **Representative** | **Same source** | **Exposure** | **Outcome absent at baseline** | **Comparability** | **Outcome assessment** | **Follow-up long enough** | **Follow-up complete** | **Stars** | **Overall Risk** |
| --- | --- | --- | --- | --- | --- | --- | --- | --- | --- | --- | --- |
| Plaudis | 2012 | ★ | ★ | ★ | ★ | — | ★ | ★ | ★ | 7 | High |
| Li | 2007 | ★ | ★ | ★ | ★ | — | ★ | ★ | ★ | 7 | High |
| Wu | 2009 | ★ | ★ | ★ | ★ | — | ★ | ★ | ★ | 7 | High |
| Liu | 2015 | ★ | ★ | ★ | ★ | — | ★ | ★ | ★ | 7 | High |
| Liu | 2025 | ★ | ★ | ★ | ★ | ★★ | ★ | ★ | ★ | 9 | Low |
| Jin | 2018 | ★ | ★ | ★ | ★ | — | ★ | ★ | ★ | 7 | High |
| Dou | 2024 | ★ | ★ | ★ | ★ | — | ★ | ★ | ★ | 7 | High |

1 star = criterion met; comparability can receive 0–2 stars. Overall risk was translated to Low / Moderate / High.
