# Supplementary material for: Safety and efficacy of microbiota-directed adjunctive therapy in acute pancreatitis: a systematic review and meta-analysis of prebiotics, probiotics, and synbiotics
Source: Front Med (Lausanne). 2026 Sep 11;13:1873327. doi: 10.3389/fmed.2026.1873327 (PMC13612259; doi:10.3389/fmed.2026.1873327)
Supplement: Supplementary file 6 [file Table_5.DOCX]

**Supplementary Table 5. Assessment of small-study effects for the primary outcomes.** Egger’s test, Begg’s test, and trim-and-fill analyses for hospital stay and mortality in the full-analysis and sensitivity-analysis sets.

| **Outcome** | **Analysis set** | **Egger test, p** | **Begg test, p** | **Trim-and-fill direction** | **Imputed studies, n** | **Observed pooled estimate** | **Adjusted pooled estimate** |
| --- | --- | --- | --- | --- | --- | --- | --- |
| Hospital stay | Full analysis | 0.4476 | 0.0435 | Right | 3 | MD -0.816 (95% CI -0.870 to -0.763) | MD -0.816 (95% CI -0.870 to -0.763) |
| Hospital stay | Sensitivity analysis | 0.9880 | 0.2831 | Right | 1 | MD -0.689 (95% CI -0.744 to -0.633) | MD -0.689 (95% CI -0.744 to -0.633) |
| Mortality | Full analysis | 0.4248 | 0.7703 | Right | 1 | log RR -0.453 (95% CI -0.892 to -0.015) | log RR -0.412 (95% CI -0.845 to 0.021) |
| Mortality | Sensitivity analysis | 0.7537 | 0.8370 | Right | 0 | log RR -0.303 (95% CI -0.900 to 0.294) | log RR -0.303 (95% CI -0.900 to 0.294) |
