# Supplementary material for: Safety and efficacy of microbiota-directed adjunctive therapy in acute pancreatitis: a systematic review and meta-analysis of prebiotics, probiotics, and synbiotics
Source: Front Med (Lausanne). 2026 Sep 11;13:1873327. doi: 10.3389/fmed.2026.1873327 (PMC13612259; doi:10.3389/fmed.2026.1873327)
Supplement: Supplementary file 7 [file Table_6.docx]

**Table S6. Study-level safety and adverse-event summary**

| **Study** | **Bacteremia/Fungemia** | **Bowel / intestinal ischemia** | **GI intolerance / diarrhea / vomiting** | **Other adverse events / complications** |
| --- | --- | --- | --- | --- |
| Karakan (2007) | NR | NR | NR | NR |
| Liu (2025) | NR | NR | NR | NR |
| Oláh (2002) | NR | NR | NR | NR |
| Li (2007) | NR | NR | NR | NR |
| Besselink (2008) | NR | 9/152 vs 0/144 | NR | NR |
| Qin (2008) | NR | NR | NR | NR |
| Cui (2009) | NR | NR | NR | NR |
| Wu (2009) | NR | NR | NR | NR |
| Lata (2010) | NR | NR | NR | NR |
| Sharma (2011) | NR | NR | NR | NR |
| Cui (2013) | NR | NR | NR | NR |
| Wang (2013) | NR | NR | NR | NR |
| Li (2014) | NR | NR | NR | peripancreatic infection: 5/27 vs 8/28; upper gi bleeding: 2/27 vs 6/28; renal failure: 1/27 vs 3/28 |
| Zhu (2014) | NR | 2/20 vs 0/19 | NR | NR |
| Liu (2015) | NR | NR | NR | NR |
| Wang (2017) | NR | NR | NR | total adverse reactions: 0/35 vs 0/36 |
| Wu (2017) | NR | NR | NR | NR |
| Fang (2018) | NR | NR | NR | total adverse reactions: 5/34 vs 3/34 |
| Jin (2018) | NR | NR | NR | pancreatic pseudocyst: 6/30 vs 5/30; pancreatic abscess: 3/30 vs 5/30; pancreatic calcification: 2/30 vs 1/30 |
| Liao (2020) | NR | NR | NR | NR |
| Wan (2021) | NR | NR | abdominal discomfort: 12/64 vs 9/64; nausea: 3/64 vs NR; abdominal pain: 3/64 vs NR; flatulence: 5/64 vs NR | probiotic-related serious AE: 0/64 vs 0/64 |
| Xiao (2022) | NR | NR | NR | NR |
| Dou (2024) | NR | NR | diarrhea: 1/82 vs 1/78; vomiting nausea: 2/82 vs 1/78 | total adverse reactions: 4/82 vs 2/78; rash: 1/82 vs 0/78 |
| Zhao (2024) | NR | NR | NR | total adverse reactions: 0/34 vs 2/32 |
| Ma (2025) | NR | NR | NR | total complications: 7/30 vs 11/30 |
| Cheng (2026) | NR | NR | NR | NR |
| He (2026) | Fungemia endpoint mentioned; separate count NR | NR | NR | NR |
| Oláh (2007) | NR | NR | NR | NR |
| Plaudis (2012) | NR | NR | NR | NR |
| Rohith (2022) | Bacteremia: 5/39 vs 13/37 | NR | NR | pneumonia: 9/39 vs 15/37; urinary infection: 5/39 vs 5/37 |

**Abbreviations:** NR = not reported or not separately extractable
